# Dihydroartemisinin-Piperaquine and Artemether-Lumefantrine for Treating Uncomplicated Malaria in African Children: A Randomised, Non-Inferiority Trial

**DOI:** 10.1371/journal.pone.0007871

**Published:** 2009-11-17

**Authors:** Quique Bassat, Modest Mulenga, Halidou Tinto, Patrice Piola, Steffen Borrmann, Clara Menéndez, Michael Nambozi, Innocent Valéa, Carolyn Nabasumba, Philip Sasi, Antonella Bacchieri, Marco Corsi, David Ubben, Ambrose Talisuna, Umberto D'Alessandro

**Affiliations:** 1 Barcelona Centre for International Health Research (CRESIB), Hospital Clínic, Institut d'Investigacions Biomèdiques August Pi i Sunyer (IDIBAPS), Universitat de Barcelona, Barcelona, Spain; 2 Manhiça Health Research Centre (CISM), Manhiça, Mozambique; 3 Tropical Disease Research Centre, Ndola, Zambia; 4 Centre Muraz, Bobo-Dioulasso, Burkina Faso, IRSS/DRO, Bobo-Dioulasso, Burkina Faso; 5 Epicentre/MSF, Mbarara, Uganda; 6 Kenya Medical Research Institute, Kilifi, Kenya; 7 University of Heidelberg, Heidelberg, Germany; 8 Department of Clinical Pharmacology, Muhimbili University of Health and Allied Sciences, Dar es Salaam, Tanzania; 9 Sigma Tau Industrie Farmaceutiche Riunite, Pomezia, Rome, Italy; 10 Medicines for Malaria Venture, Geneva, Switzerland; 11 Prince Leopold Institute of Tropical Medicine, Antwerp, Belgium; University of Cape Town, South Africa

## Abstract

**Background:**

Artemisinin combination therapies (ACTs) are currently the preferred option for treating uncomplicated malaria. Dihydroartemisinin-piperaquine (DHA-PQP) is a promising fixed-dose ACT with limited information on its safety and efficacy in African children.

**Methodology/Principal Findings:**

The non-inferiority of DHA-PQP versus artemether-lumefantrine (AL) in children 6–59 months old with uncomplicated *P. falciparum* malaria was tested in five African countries (Burkina Faso, Kenya, Mozambique, Uganda and Zambia). Patients were randomised (2∶1) to receive either DHA-PQP or AL. Non-inferiority was assessed using a margin of −5% for the lower limit of the one-sided 97.5% confidence interval on the treatment difference (DHA-PQP vs. AL) of the day 28 polymerase chain reaction (PCR) corrected cure rate. Efficacy analysis was performed in several populations, and two of them are presented here: intention-to-treat (ITT) and enlarged per-protocol (ePP). 1553 children were randomised, 1039 receiving DHA-PQP and 514 AL. The PCR-corrected day 28 cure rate was 90.4% (ITT) and 94.7% (ePP) in the DHA-PQP group, and 90.0% (ITT) and 95.3% (ePP) in the AL group. The lower limits of the one-sided 97.5% CI of the difference between the two treatments were −2.80% and −2.96%, in the ITT and ePP populations, respectively. In the ITT population, the Kaplan-Meier estimate of the proportion of new infections up to Day 42 was 13.55% (95% CI: 11.35%–15.76%) for DHA-PQP vs 24.00% (95% CI: 20.11%–27.88%) for AL (p<0.0001).

**Conclusions/Significance:**

DHA-PQP is as efficacious as AL in treating uncomplicated malaria in African children from different endemicity settings, and shows a comparable safety profile. The occurrence of new infections within the 42-day follow up was significantly lower in the DHA-PQP group, indicating a longer post-treatment prophylactic effect.

**Trial Registration:**

Controlled-trials.com ISRCTN16263443

## Introduction

Artemisinin-based combination therapies (ACTs) are highly efficacious and fast acting antimalarial medicines. The World Health Organization (WHO) recommends their use for treating uncomplicated malaria [1]. In Africa, their introduction on a wide scale began in 2003 and currently most African countries have adopted or are using ACTs as first or second line treatments, either artesunate-amodiaquine or artemether-lumefantrine (AL) [2], available as co-formulations produced under GMP, though the former is also used as a co-blistered or non-co-formulated product. The co-formulation of dihydroartemisinin (DHA), the active metabolite of artemisinin derivatives, with piperaquine (PQP), a bisquinoline structurally close to chloroquine, seems to be a promising combination and a good alternative to AL, whose optimal use in the public health system is challenged by the twice-daily dosing scheme and the need for co-administration with fatty food [3], necessary for improving the absorption of lumefantrine. DHA-PQP provides a simpler dosage scheme (a single daily dose over 3 days) than AL and is generally administered without specific food instructions, though recent data indicate that co-administration with fat (milk, biscuit, or other food) increases bio-availability of piperaquine and possibly efficacy [4].

Several trials [5]–[8] have assessed DHA-PQP safety, efficacy and effectiveness [9], mostly in Asia, reporting an efficacy of about 90% over 28–63 days [8]. There is little information on the safety and efficacy of DHA-PQP in African children, as only a few single-centre trials [10]–[13] have been done in Africa. DHA-PQP is registered in several countries in Africa and South-East Asia and has been widely used in Vietnam or Cambodia, though these formulations are not manufactured according to internationally recognised GMP. In 2005, a public-private partnership programme funded by the Medicines for Malaria Venture (MMV) and led by the Italian Company Sigma-Tau I.F.R. SpA (Rome) in collaboration with the University of Oxford was set up to fill the gaps needed for the international registration of DHA-PQP. This included a phase III, randomized multicentre trial to test the non-inferiority of DHA-PQP compared with AL in treating uncomplicated malaria in African children.

## Methods

The protocol for this trial and supporting non-inferiority adapted [14] CONSORT checklist are available and annexed as supporting information; see Protocol S1 and Checklist S1.

### Ethical Considerations and Patient Safety

The study protocol was approved by the Institutional Review Board of the Institute of Tropical Medicine, Antwerp, the Ethical Committee of the Antwerp University Hospital, the University of Heidelberg Ethics Committee and by the National Ethics Review Committee or Institutional Review Board at each trial site. The trial was conducted under the provisions of the Declaration of Helsinki and in accordance with Good Clinical Practices guidelines set up by the International Conference on Harmonization. A Study Steering Committee, a Data Monitoring Committee and a Clinical Development Committee were created prior to the beginning of the trial, and worked independently to harmonise and monitor the study. The trial was registered prior to the enrolment of the first patient in the International Standard Randomized Controlled Trials Register, number ISRCTN 16263443, at http://www.controlled-trials.com/isrctn.

### Study Design, Sites and Concealment of Patient Allocation

Between August 2005 and July 2006, a randomised, open-label, multicentre clinical trial was carried out in five African sites (Nanoro, Burkina Faso; Kilifi, Kenya; Manhiça, Mozambique; Mbarara, Uganda; and Ndola, Zambia). Characteristics of the five sites are summarized in Table 1. [ONLINE PUBLICATION ONLY]

**Table 1:** Basic characteristics of the 5 African sites (online publication only).

	Nanoro, Centre Muraz (Burkina Faso)	Kilifi, KEMRI (Kenya)	CISM, Manhiça (Mozambique)	Epicentre, Mbarara (Uganda)	TDRC, Ndola (Zambia)
Characteristics of the area	Rural	Rural	Rural	Rural	Periurban
Malaria endemicity	Mesoendemic	Mesoendemic	Mesoendemic	Mesoendemic	Mesoendemic
Seasonality	High transmission between June and December	Perennial, with two peak seasons: Jul-Sep; Dec-Jan	Perennial with marked seasonality (Oct-April)	Perennial with two peaks: April and October	High transmission between November and May
Entomological Inoculation rate (EIR)	100 to 160 (2003)	22 to 53^1^	38 (2002)^2^	Not available	Not available
Site area under Demographic surveillance system (DSS)	No	Yes	Yes	No	No
ITNs coverage	<10%	Subsidised available	<10%	11,4%	Approximately 30%
First line treatment at the time of the study	Amodiaquine-artesunate or AL	SP, and then AL	Amodiaquine-SP	AL	AL
Documented resistance to chloroquine	35%	60%	69%^3^	81%^4^	60%
Dates start patients' recruitment/end follow up	16 Aug 2005/18 Jan 2006	22 Sep 2005/14 July 2006	14 Nov 2005/10 July 2006	17 Oct 2005/11 July 2006	16 Nov 2005/10 July 2006

1 Mbogo CM, Mwangangi JM, Nzovu J, Gu W, Yan G, Gunter JT, et al. Spatial and temporal heterogeneity of Anopheles mosquitoes and Plasmodium falciparum transmission along the Kenyan coast. Am J Trop Med Hyg. 2003 Jun; 68(6):734–42.

2 Alonso PL, Sacarlal J, Aponte JJ, Leach A, Macete E, Milman J, et al. Efficacy of the RTS,S/AS02A vaccine against Plasmodium falciparum infection and disease in young African children: randomised controlled trial. Lancet. 2004 Oct 16; 364(9443):1411–20.

3 Abacassamo F, Enosse S, Aponte JJ, Gomez-Olive FX, Quinto L, Mabunda S, et al. Efficacy of chloroquine, amodiaquine, sulphadoxine-pyrimethamine and combination therapy with artesunate in Mozambican children with uncomplicated malaria. Trop Med Int Health. 2004 Feb; 9(2):200–8.

4 Legros D, Johnson K, Houpikian P, Makanga M, Kabakyenga JK, Talisuna AO, et al. Clinical efficacy of chloroquine or sulfadoxine-pyrimethamine in children under five from south-western Uganda with uncomplicated falciparum malaria. Trans R Soc Trop Med Hyg. 2002 Mar-Apr; 96(2):199–201.

Children 6–59 months old attending the health facilities with uncomplicated malaria were included in the study if they fulfilled the following inclusion criteria: body weight >5 kg; microscopically confirmed *Plasmodium falciparum* mono-infection with asexual parasite densities between 2,000 and 200,000/µl; fever (axillary temperature ≥37.5°C) or history of fever in the preceding 24 h. Patients were not recruited if they met at least one of the following exclusion criteria: severe malaria [15], or other danger signs; acute malnutrition (weight for height <70% of the median National Center for Health Statistics/WHO reference) or any other concomitant illness or underlying disease; contra-indication to receive the trial drugs or ongoing prophylaxis with drugs having antimalarial activity. Patients satisfying the inclusion/exclusion criteria were enrolled if the parent/guardian signed a detailed written informed consent.

Patients were individually randomised according to a 2∶1 (DHA-PQP∶AL) scheme, so as to have more patients in the DHA-PQP arm to provide better estimates for its cure rates and more cases for the integrated safety data base. A randomisation list stratified by country was generated by an independent off site contract research organisation (CRO), with each treatment allocation concealed in opaque sealed envelopes that were opened only after the patient's recruitment.

Both drugs were administered under direct supervision during 3 consecutive days, according to the patient's body weight. AL (Coartem™, Novartis, Switzerland) was administered twice a day (at enrolment and at 8, 24, 36, 48 and 60 h) according to the following dosage: weight 5–14 kg: one tablet per dose; weight 15–24 kg: two tablets per dose; weight 25–34 kg: three tablets per dose. DHA-PQP (Eurartesim™, Sigma-Tau, Italy) was given once daily, at the standard dosage of 2.25 mg/kg and 18 mg/kg per dose of DHA and PQP, respectively, rounded up to the nearest half tablet. To facilitate the correct dosing of DHA-PQP, two formulations were used (DHA 20 mg + PQP 160 mg and DHA 40 mg + PQP 320 mg). In case of vomiting, a full dose was repeated if this occurred within the first half an hour, or half a dose if it occurred between 30 minutes and 1 h. AL was administered concomitantly with milk (as recommended by the manufacturer) while for DHA-PQP no specific instructions regarding co-administration with food were given. For infants, drugs were crushed, mixed with water and administered as slurry.

Both patient allocation to the different analysis populations and assessment of the primary end-point were made by staff blinded to the treatment assignment and before availability of the PCR results.

### Treatment Follow-Up, Clinical and Laboratory Procedures

All children were kept at the health facility for the 3-day dosing period. The mother/guardian was asked to return with the child for scheduled visits on days 7, 14, 21, 28, 35 and 42 post-treatment, or if any symptoms occurred. Field workers traced patients missing any visit. For each visit, a physical examination was performed by the study clinicians, vital signs were recorded, and axillary temperature measured with an electronic thermometer. Adverse events and serious adverse events were recorded and monitored throughout the study. A 12-lead electrocardiogram (ECG) was performed at enrolment and repeated on days 2 and 7 to assess any QT/QTc interval prolongation. Any ECG abnormality detected at enrolment requiring urgent management was considered an exclusion criterion. All ECG records were transmitted daily online to a central cardiologist (Paris, France) who interpreted them in a blinded manner, and feedback was sent to the sites as soon as available. The QTc interval (ms) was evaluated after correcting for the heart rate with Bazett's or Fridericia's formulae and classified according to the following categories: Normal <430 ms; Borderline: 431–450 ms; Prolonged >450 ms.

The study was supervised by monthly monitoring visits. Rescue treatment for recurrent parasitaemia was according to local national guidelines. All participants, with the exception of those in Kilifi, received a free insecticide-treated bed net at recruitment.

Capillary or venous blood was taken at every visit. Thick and thin blood films were prepared, dried and Giemsa-stained, and parasite density estimated by counting the number of asexual parasites in 200 white blood cells (WBC), assuming a standard WBC count of 8,000/µl. Quality control was performed in blind conditions on 20% of all the slides at a central laboratory. Samples for haematology (full blood count) and biochemistry (liver and renal function) were taken at enrolment, at days 3, 28 and 42, and at any other visit if judged necessary by the clinician. For PCR analysis, three blood spots were collected on filter paper (Whatmann 3 MM) at enrolment and at any visit after day 7. Each filter paper was dried and individually stored in a plastic bag containing silica gel. All filter papers were subsequently transferred to the Institute of Tropical Medicine (Antwerp, Belgium) where centralised genotyping was conducted. Purification of DNA was conducted as previously described [16]. Three polymorphic genetic markers MSP1, MSP2 and GluRP were used to distinguish recrudescence from new infections [17], [18]. Recrudescence was defined as at least one identical allele for each of the three markers in the pre-treatment and post-treatment samples. New infections were diagnosed when all alleles for at least one of the markers differed between the two samples. All gels were re-read under blinded conditions by an independent expert (National Museum of Natural History, Paris, France). In addition, 20% of the filter papers were re-analysed and assessed by an independent laboratory (Shoklo Malaria Research Unit, Mae Sot, Thailand).

### Outcome Classification

The primary endpoint was the PCR-corrected adequate clinical and parasitological response (ACPR) at day 28; secondary efficacy outcomes included PCR-corrected cure rates at days 14 and 42, PCR-uncorrected cure rates at days 14, 28 and 42; parasite and fever clearance times, presence and clearance of gametocytes, and haemoglobin (Hb) changes from baseline to day 28. All standard safety outcomes such as incidence of adverse events, changes from baseline on haematology and clinical chemistry parameters, ECG findings and vital sign variation during the study were also evaluated.

Treatment outcome was analysed in two ways. The first, based on a pre-defined (in the protocol) procedure further developed with the Data Monitoring and the Clinical Development Committees, complemented the WHO definitions (see below) with a set of rules allowing the evaluation of each individually randomised patient, e.g. patients having taken not-allowed anti-malarial drugs or with halfway missing data such as blood parasitaemia (Table 2). Such an approach was defined as primary because it was deemed in line with the requirements of the most stringent regulatory authorities. All cases not strictly matching the WHO definitions and/or the described procedure were reviewed individually at the data review meetings in blind conditions. The second approach, based on the standard definitions of early/late clinical and parasitological failure (World Health Organization) [19], was used to allow comparison with previously published results. Accordingly, true treatment failure (TTF) was defined as the sum of the early and late (either LPF or LCF) treatment failures occurring until Day 13 (recrudescence by default) and the late treatment failures from Day 14 onwards classified as recrudescence by PCR analysis.

**Table 2:** Day 28 and Day 42 uncorrected ACPR (steps 1–11) and PCR-corrected ACPR (steps 1–16) in the different populations of analysis.

Step	Event to be assessed	ePP	ITT
1	Withdrawal BEFORE OR AT D28: any reason except lost to follow-up (LFU)	Depending on reason, a patient can be: Excluded or Failure	Failure
2	Withdrawal BEFORE OR AT D28: LFU	Excluded	Failure
3*	Withdrawal AFTER D28: any reason except LFU	Failure	Failure
4*	Withdrawal AFTER D28: LFU	Failure	Failure
5	ETF, LCF, and LPF in accordance with the WHO criteria	Failure	Failure
6**	Presence of major protocol violations	Excluded	No effect
7**	Occurrence of adverse events highlighting recurrence of malaria	Failure	Failure
8**	Presence of missing parasitaemia at two or more consecutive scheduled visits or presence of an isolated missing parasitaemia not preceded and followed by a negative parasitaemia	Failure	Failure
9**	Administration of drugs with a known or suspected anti-malaria action as rescue treatment	Failure	Failure
10**	Administration of drugs with a known or suspected anti-malaria action as non rescue treatment	Excluded	Failure
11**	Administration of anti-malarial drugs for *P. vivax*, *P. malariae*, or *P. ovale* during the course of the study in patients not classified as ETF/LTF	Failure with new infection	Failure with new infection
12	PCR not done IN (DAY 4–DAY 13)	Recrudescence	Recrudescence
13	PCR: non interpretable or missing or not done IN (DAY 14–D28)	Excluded	Recrudescence
14**	PCR: non interpretable or missing or not done AFTER D28	Rule***	Recrudescence
15	PCR = new infection or uncorrected ACPR = Failure with new infection	Success	Success
16	PCR = recrudescence	Recrudescence	Recrudescence

*For the Day 42 endpoint.

**All such cases were individually revised at the Blind Data Review meeting. Protocol violations were pre-defined.

***Result “recrudescence” or “new infection” was assigned according to the ratio between these outcomes in the patients with a valid PCR result at the corresponding time point and within each treatment group, separately considered.

### Statistical Analysis

Several populations were defined for the analysis and two of them (intention-to-treat, ITT, and enlarged per-protocol, ePP), despite not being the primary ones as defined by the protocol, are presented here on the basis of their comparability with the populations discussed in previous published studies, their clinical relevance and the fact that conclusions are similar on all considered populations. The ITT included all randomised patients having taken at least one dose of the study treatments. The ePP population included all randomised patients fulfilling the protocol eligibility criteria, having taken at least 80% of the study medication when not previously classified as early treatment failures, completing the day 28 assessment and having an evaluable PCR in case of recurrent parasitaemia. Table 2 provides details for patient classification in these two populations.

Primary efficacy analysis was based on a 97.5% (one-sided) confidence interval (CI) computed on the difference between the day 28 PCR-corrected cure rates (defined as in Table 2) of DHA-PQP and AL. To prove non-inferiority, the lower limit of this CI was to be within −5%, the pre-established non-inferiority margin. The Wald method (without continuity correction) was used to compute the CI, as this method was known to provide control of type I error around the nominal level for the 2∶1 allocation, and also in the context of a hypothesis test of non-inferiority [20]. PCR-corrected and uncorrected cure rates at the other time points were assessed similarly. TTF was estimated with the Kaplan-Meier method as suggested by WHO, in the ePP population. Patients withdrawing the study, with a new infection, or with a non-interpretable or missing PCR were censored at the withdrawal or PCR sampling time.

Survival analysis was applied also to the new infections. In this case censoring was applied to recrudescences.

Cure rates were also stratified by country and age (age groups: ≤12 months; >12 months), though the study was not powered for proving efficacy within each country or age group. The Breslow-Day test, or logistic regression when the former was not applicable, was used to assess homogeneity across countries and age groups. For exploratory testing, categorical variables were compared using χ^2^ or Fisher's exact test, and continuous variables using the Student t-test for independent samples.

Rates of person-gametocyte-weeks for measuring gametocyte carriage and transmission potential were calculated as the number of weeks in which blood slides were positive for gametocytes divided by the total number of follow-up weeks and expressed per 1,000 person-weeks.

All safety variables were analysed in the ITT population.

### Sample Size Calculation

This study was designed as a non-inferiority trial. Assuming 80% statistical power, a one-sided α level of 2.5%, and adopting an unequal 2∶1 randomisation ratio, 1,500 patients (1000 DHA-PQP, 500 AL) were needed to show that the difference of the day 28 PCR-corrected cure rates between DHA-PQP and AL was within −5%, assuming a response rate for AL of at least 92%.

## Results

### Trial Profile and Baseline Characteristics

**Figure 1:**
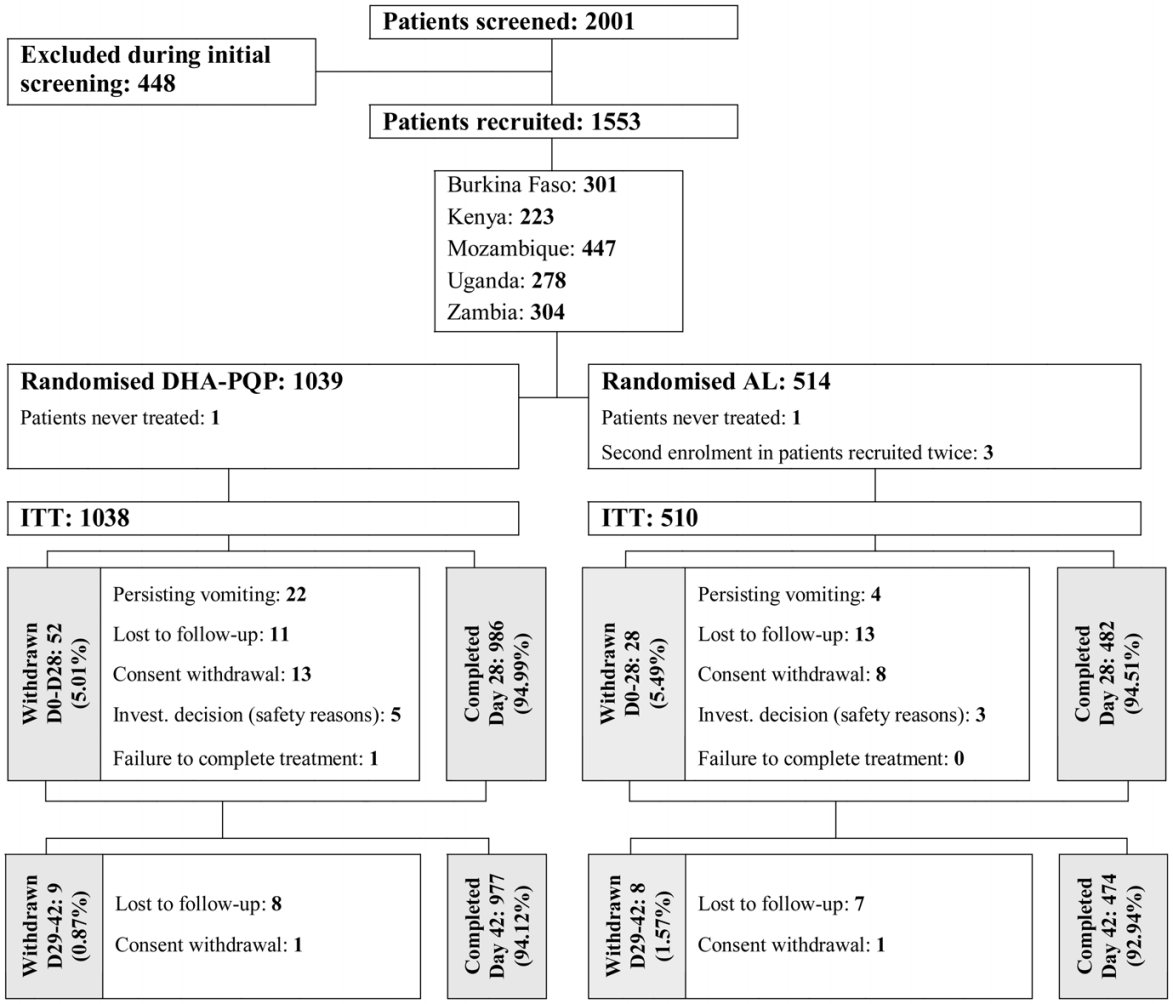
Trial profile.

Overall, 2,001 patients were screened, and 1,553 recruited and randomised to receive the study drugs (1,039 DHA-PQP and 514 AL) (Figure 1). Five patients were excluded from all analyses: one child in each treatment group who did not receive any treatment and three children in the AL group who were recruited twice (only data for the first recruitment were retained). A total of 1,548 patients were considered for the ITT population and the safety analysis, and the ePP population consisted of 1,425 patients. The attrition rate of the ePP population as compared to the ITT was approximately 8% and was due to lost-to-follow-up (∼2%) or major protocol violations (∼6%). These proportions were equally distributed between treatments (data not shown).

Randomisation generated comparable groups between countries and overall (Table 3).

**Table 3:** Baseline characteristics (ITT population).

Variable	DHA-PQP (N = 1038)	AL (N = 510)
Gender M/F (%M/%F)	525/513 (50.1/49.4)	281/229 (55.1/44.9)
Age in years (mean±SD)	2.42±1.14	2.43±1.16
Weight in kg (mean±SD)	11.19±2.55	11.28±2.67
Fever (n (%))	624 (60.12)	307 (60.20)
Temperature in °C (mean±SD)	37.88±1.22	37.86±1.18
Parasite density (geometric mean)	24557	25884
Presence of Gametocytes (n (%))	122 (11.75)	66 (12.94)
Hb in g/L (mean±SD)	89.23±18.15	90.59±18.20
Anaemia ( = Hb<7 g/dL) (n (%))	141 (13.58)	63 (12.35)
Leucocytes in 10̂9/L (mean±SD)	9.62±4.15	9.59±3.94
Platelets in 10̂9/L (mean±SD)	182.84±108.70	181.59±106.74
Splenomegaly (n (%))	41 (3.95)	19 (3.73)
Hepatomegaly (n (%))	6 (0.58)	3 (0.59)
ALAT in IU/L (mean±SD)	34.08±61.34	31.08±36.23
Bilirubin in mg/dl (mean±SD)	0.97±1.04	0.94±0.81
Creatinine in U/L (mean±SD)	40.96±17.91	41.16±19.17

### Efficacy Results

**Figure 2:**
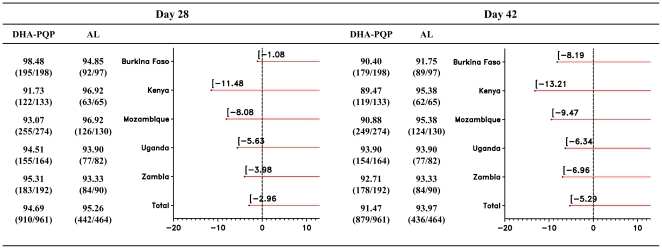
PCR-corrected Adequate Clinical and Parasitological Response (ACPR) (ePP population) by country and by time point.

DHA-PQP was as efficacious as AL. The day 28 PCR-corrected cure rate was 90.4% (ITT) and 94.7% (ePP) in the DHA-PQP group, and 90.0% (ITT) and 95.3% (ePP) in the AL group (ITT: p = 0.820; ePP: p = 0.650). The lower limits of the one-sided 97.5% CIs on the differences between the two treatments were −2.80% and −2.96%, in the ITT and ePP populations, respectively (Table 4 and Figure 2). The analyses in the other populations and all sensitivity analyses confirmed the robustness of these results. The day 42 PCR-corrected cure rates were lower than those at day 28 but similar for the two treatments for the ITT population (Table 4). However, the lower limit of the one-sided 97.5% CI on the cure rate difference for the ePP population was −5.29%, a value slightly below the pre-established non-inferiority margin (Table 4 and Figure 2). The details of the classification of patients for the PCR-corrected response both in the ITT and ePP populations are presented in Table 4 for both the analyses at day 28 and day 42. The percentage of recrudescent infections and new infections, as detected by PCR at or before day 28, was lower in the DHA-PQP group with respect to the AL group, while there were more withdrawals and/or treatment failures at or before day 14 in the DHA-PQP group compared with the AL group (Table 4). At day 42, the findings were similar with slightly more recrudescent infections in the DHA-PQP group.

**Table 4:** PCR-Corrected and Uncorrected Adequate Clinical and Parasitological Response (ACPR) by time point in ITT and ePP Population.

	Day 28			Day 42		
	DHA-PQP	AL	Lower Limit of one-sided 97.5% CI on difference	DHA-PQP	AL	Lower Limit of one-sided 97.5% CI on difference
**PCR-Corrected Cure Rate (n (%)) in ITT**	938 (90.37)	459 (90.00)	−2.80	895 (86.22)	442 (86.67)	−4.06
**Uncorrected Cure Rate (n (%)) in ITT**	910 (87.67)	391 (76.67)	6.82	769 (74.08)	330 (64.71)	4.45
**Total number of failures in ITT (PCR-uncorrected)**	128 (12.33)	119 (23.33)		269 (25.92)	180 (35.29)	
Recrudescences by PCR	14 (1.35)	11 (2.16)		41 (3.95)	17 (3.33)	
Recrudescences due to informative withdrawals (including LFU) or failure before D14 (PCR not needed)	65 (6.26)	26 (5.10)		65 (6.26)	26 (5.10)	
Recrudescences imputed (PCR missing, indet., not done)	21 (2.02)	14 (2.75)		37 (3.56)	25 (4.90)	
New Infection by PCR	27 (2.60)	64 (12.55)		122 (11.75)	105 (20.59)	
New Infection ≠ from Plasmodium Falciparum	1 (0.10)	4 (0.78)		4 (0.39)	7 (1.37)	
**PCR-Corrected Cure Rate (n (%)) in ePP**	910 (94.69)	442 (95.26)	−2.96	879 (91.47)	436 (93.97)	−5.29
**Uncorrected Cure Rate (n (%)) in ePP**	884 (91.99)	376 (81.03)	6.99	746 (77.63)	319 (68.75)	3.90
**Total number of failures in ePP (PCR-uncorrected)**	77 (8.01)	88 (18.97)		215 (22.37)	145 (31.25)	
Recrudescences by PCR	14 (1.46)	11 (2.37)		41 (4.27)	16 (3.45)	
Recrudescences due to informative withdrawals or failure before D14 (PCR not needed)	37 (3.85)	11 (2.37)		37 (3.85)	11 (2.37)	
Recrudescences imputed (rule for missing PCR)	0	0		4 (0.42)	1 (0.22)	
New Infections imputed (rule for missing PCR)	0	0		11 (1.14)	8 (1.72)	
New Infection by PCR	25 (2.60)	62 (13.36)		118 (12.28)	102 (21.98)	
New Infection ≠ from Plasmodium Falciparum	1 (0.10)	4 (0.86)		4 (0.42)	7 (1.51)	

Note: In ITT, percentages are based on N = 1038 (DHA-PQP) and N = 510 (AL); in ePP, percentages are based on N = 961 (DHA-PQP) and N = 464 (AL).

The day 28 PCR-corrected cure rates in infants (6–11 months-old) were similar to those in older children and above 90% in both treatment groups (ITT: DHA-PQP 90.70%, AL 92.65%, p = 0.643, 97.5% CI>−9.92%).

**Figure 3:**
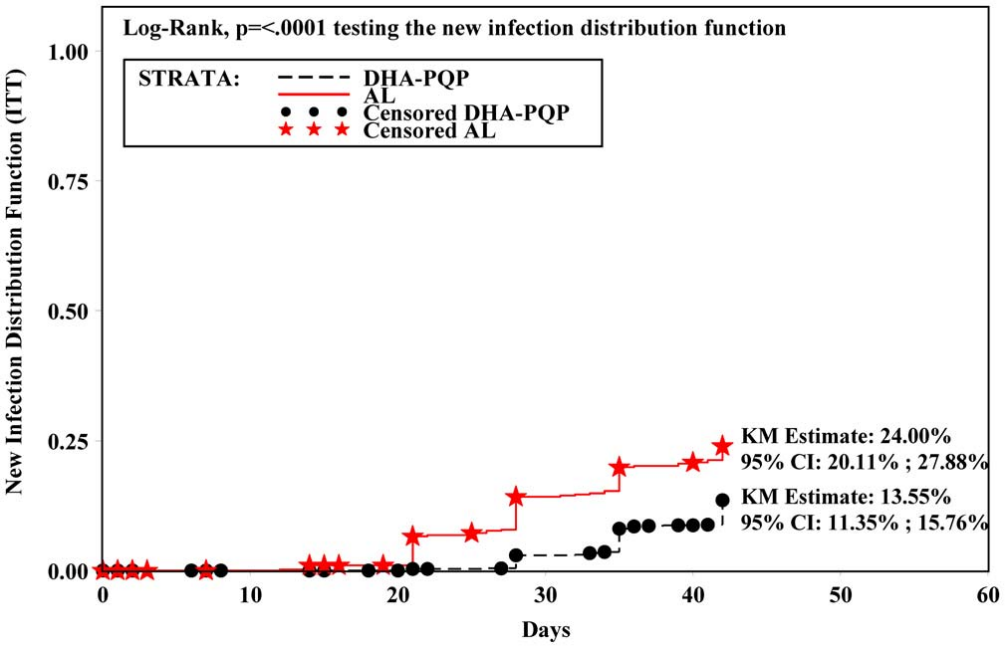
Kaplan Meier curve showing the cumulative proportion until day 42 of children with new infections (ITT population).

The uncorrected cure rates were significantly higher in the DHA-PQP group, both at day 28 (ITT: DHA-PQP 87.7% vs. AL 76.7%, p<0.001, 97.5% CI>6.82%; ePP: DHA-PQP 91.99% vs. AL 81.03%, p<0.001, 97.5% CI>6.99%) and at day 42 (ITT: DHA-PQP 74.08% vs. AL 64.71%, p<0.001, 97.5% CI>4.45%; ePP: DHA-PQP 77.63% vs. AL 68.75%, p<0.001, 97.5% CI>3.90%). This was mainly due to fewer late failures later classified as new infections in the DHA-PQP as compared to the AL arm. In the ITT population, new infections until day 42 occurred significantly less in the DHA-PQP group (Kaplan-Meier estimate: 13.55%; 95% CI: 11.35%–15.76%) than in the AL group (Kaplan-Meier estimate: 24.00%; 95% CI: 20.11%–27.88%) (Figure 3). Similar results were obtained in the ePP population (data not shown).

When the day 28 PCR-corrected cure rates were analysed by country, the heterogeneity test was borderline significant at the 10% level only in the ePP population (ITT: p = 0.324; ePP: p = 0.082), suggesting some minor differences among sites (Figure 2). However, the CIs adjusted by country were almost identical to the unadjusted ones (data not shown). At day 42, the heterogeneity test was not statistically significant in either the ITT or the ePP populations (ITT: p = 0.582; ePP: p = 0.703). Heterogeneity across countries was more marked for the uncorrected cure rates at day 28 (ITT and ePP: p<0.001), while at day 42 the heterogeneity test was borderline significant only in the ePP population (ITT: p = 0.186; ePP: p = 0.051). However, with the exception of Kenya, such differences on the uncorrected cure rates were of a quantitative type, i.e. rather in the size of the treatment effect across countries, not in its direction, always favouring DHA-PQP.

When considering the WHO standard definition of TTF, the two treatment groups were similar at day 28 (Kaplan-Meier estimate in ePP: DHA-PQP 3.78% [95%CI: 2.57%–5.00%] vs AL 3.19% [95%CI: 1.54%–4.84%], p = 0.528), while at day 42 TTF tended to be lower in the AL group (Kaplan-Meier estimate in ePP: DHA-PQP 6.86% [95%CI: 5.22%–8.50%] vs AL 4.52%, [95%CI: 2.52%–6.51%], p = 0.119).

Parasite clearance was rapid in both treatment groups (Kaplan-Meier estimate of median time was 2 days in each group, in both populations). About 60% of patients had fever at baseline while at day 2 more than 97% of patients were afebrile in both treatment groups. Gametocyte prevalence at recruitment was similar in both study arms (ITT: DHA-PQP 11.75%; AL 12.94%, p = 0.501; ePP: DHA-PQP 11.55%; AL 13.36%, p = 0.326). However, gametocyte carriage measured as rate of person-gametocyte-weeks was significantly higher in the DHA-PQP group than in the AL group, both for the ITT (DHA-PQP: 43.97/1,000; AL: 21.43/1,000; p = 0.005) and the ePP (DHA-PQP: 42.65/1,000; AL: 21.23/1,000; p = 0.006) populations.

Haemoglobin changes from baseline to day 28 were comparable between treatment groups (data not shown) while the change from baseline to the last available data was significantly higher in the DHA-PQP group than in the AL group (ITT: 17.0±18.18 g/L vs 14.27±18.54 g/L, p = 0.007; ePP: 17.19±17.96 g/L vs 15.07±18.56 g/L, p = 0.044).

### Safety Results

Both DHA-PQP and AL were well tolerated with the majority of adverse events of mild or moderate severity, and consistent with symptoms attributable to malaria (Table 5). There were no significant differences in the occurrence of events, including serious adverse events. Gastrointestinal tolerability of both drugs was similar (DHA-PQP: 207/1038, 19.9%; AL: 92/510, 18.0%), with the majority of events being mild. Cutaneous adverse events were infrequent, and mainly involved minor dermatitis or rash (DHA-PQP: 70/1038, 6.7%; AL: 29/510, 5.7%). Three patients developed urticaria (one (0.1%) in the DHA-PQP group and two (0.4%) in the AL group) and three more developed mild hypersensitivity (two (0.2%) in the DHA-PQP group and one (0.2%) in the AL group). None of them required hospitalization. Occurrence of laboratory AEs, e.g. neutropenia (DHA-PQP: 18/1038, 1.7%; AL: 12/510, 2.4%) and altered liver enzymes (ALT) (DHA-PQP: 20/1038, 1.9%; AL: 19/510, 3.7%), was similar between the two treatment groups.

**Table 5:** Summary of adverse events (ITT population).

Safety/ITT Population	DHA-PQP (N = 1038)	AL (N = 510)	p-value
At least one AE (n,%)	823 (79.29%)	411 (80.59%)	0.550
Neutropenia	18 (1.73%)	12 (2.35%)	
Vomiting	71 (6.84%)	35 (6.86%)	
Gastrointestinal disorders (including vomiting)	207 (19.94%)	92 (18.04%)	
Skin and subcutaneous tissue disorders	70 (6.74)	29 (5.69%)	
Alanine aminotransferase increased	20 (1.93%)	19 (3.73%)	
Electrocardiogram QT prolonged	26 (2.50%)	13 (2.55%)	
At least one related AE (n,%)	737 (71.00%)	368 (72.16%)	0.637
At least one SAE (n,%)	18 (1.73%)	5 (0.98%)	0.249
At least one related SAE (n,%)	15 (1.45%)	4 (0.78%)	0.332
At least one AE which caused discontinuation (n,%)	5 (0.48%)	0	0.178
At Least one SAE which caused death (n,%)	1 (0.10%)	1 (0.20%)	0.551

AE = Adverse event; SAE = Serious adverse event; Related SAE = Serious adverse event for which the investigator classifies the relationship to the study drug as unlikely, possible, probable, definitely related or whose classification is missing.

ECG was performed in more than 98% of patients at day 0 and day 2, always before the administration of the treatment (96% of patients had ECG also at day 7). In the DHA-PQP group, the proportion of patients with borderline (29.1%) and prolonged (9.1%) QTc interval at day 2 corrected by the Bazett's method was higher than in the AL group (19.8% and 6.9%) (p<0.001). However, this was not confirmed when applying the Fridericia's correction as the corresponding proportions were 1.0% and 0.2% in the DHA-PQP group and 1.2% and 0.2% in the AL group (p = 0.76). In addition, a ≥60 ms increase of the QTc interval between day 0 and day 2 (Bazett's correction) was observed in just 2.7% (DHA-PQP) and 2.0% (AL) patients; only two patients per group showed a QTc at day 2 higher than 500 ms. When considering the occurrence of the AE “Electrocardiogram QT prolonged”, similar percentages (DHA-PQP: 26/1038, 2.5%; AL: 13/510, 2.6%) were observed in the two treatment groups (Table 5).

No other difference between groups was observed during the follow up (data not shown).

Two deaths (one per group) occurred during the study. In Uganda, a 3 year-old girl died 24 h after commencing treatment with DHA-PQP. Sepsis or severe malaria was considered by the investigating clinician as the most likely cause. In Mozambique, an 18 month-old girl died 7 h after the first dose of AL. Severe malaria was considered the most likely cause of death, although other aetiologies such as sepsis, hypoglycaemia, heart conditions or bronco-aspiration could not be excluded. No autopsy could be performed in these two children and a causal relationship with the treatment could not be ruled out.

## Discussion

The fulfilment of the non-inferiority criterion on all analysis populations and the confirmation that in this study the comparator treatment had the expected efficacy [12], [21] proved that DHA-PQP is non inferior to AL in treating African children aged 6–59 months with uncomplicated malaria. The two treatments had similar safety profiles. Our study confirms the results of previous trials in Asia [8] and Africa [10]–[13] that found DHA-PQP to be as effective as other ACTs, including AL. A recent study in Papua New Guinea (PNG) reported a significantly higher cure rate (adequate clinical and parasitological response) in children treated with AL as compared to DHA-PQP [7]. The reasons for such discordant results are unclear though the authors mention the cross-resistance between chloroquine and PQP. However, PQP, though structurally related to chloroquine, has been shown to be effective *in vitro* against chloroquine-resistant strains [8], [22]. In addition, it has been suggested that the lower-than-expected DHA-PQP efficacy reported in PNG may be due to administration of the treatment without any food [4]. Indeed, PQP is highly lipid-soluble and its oral bioavailability is enhanced when given with food [23], [24], though an additional study in Vietnamese healthy volunteers reports no influence of food intake (standardised Vietnamese meal) on PQP pharmacokinetics [25]. The issue on whether to recommend the administration of DHA-PQP with a biscuit or a glass of milk remains unanswered. Though co-administration with food may improve the drug's bioavailability, it is unclear whether this will translate in a higher efficacy. In our study, DHA-PQP was given without specific instructions regarding co-administration with food but its efficacy at day 42 was over 90%, similar to that reported in a study carried out in Uganda [10] but lower than in two other African studies [12], [13]. Moreover, no clinically relevant heterogeneity was shown across the five African countries despite the high chloroquine resistance previously reported from most study sites [26]. When taking into account all recurrent infections observed during the follow up period, i.e., without the PCR correction, the cure rates for DHA-PQP were significantly better than AL, indicating a better post-treatment prophylaxis (PTP) than AL [27] and confirming that chloroquine resistance did not interfere with DHA-PQP efficacy. The significantly higher Hb change from baseline to the last available data in the DHA-PQP group is in line with this observation. Therefore, the longer PQP's elimination half-life (about 20 days) as compared to lumefantrine (4–10 days), provides a longer PTP, prevents the emergence of new infections and improves the patient's haematological recovery, despite a significant chloroquine resistance background. While this is clearly an advantage for the individual, at the population level, it may increase the risk of selecting resistant parasites among the new infection [28] and stress the need of matching the large scale deployment of DHA-PQP with the careful monitoring of resistance [29].

One hundred twenty nine infants aged 6–11 months treated with DHA-PQP responded as well to treatment as older children, though the study was not powered to confirm non-inferiority between the two treatment groups. Infants represent a special group as they are more at risk of malaria and of receiving inadequate doses of antimalarial treatments. In Papua, Indonesia, the PQP plasma concentration at day 7 was the major determinant of the therapeutic response to DHA-PQP [30]. The best cut-off for the day 7 PQP concentration predicting any treatment failure was 30 ng/ml and children had a higher risk of having lower levels [31]. Similarly, in PNG, a trend toward a lower risk of treatment failure (PCR uncorrected) and plasma PQP levels at day 7 has been reported [7], suggesting that an increase of the weight-adjusted dosage in children may be required. In our study, preliminary results on predictors of treatment failure seem to confirm the need of reviewing and possibly increasing the weight-adjusted dosage for children.

Patients treated with DHA-PQP had a significantly higher rate of person-gametocyte-weeks compared with those having received AL. This contrasts with a previous study in Papua, Indonesia, which showed no difference in gametocyte carriage between DHA-PQP and AL [30], but is in line with comparisons between DHA-PQP and mefloquine-artesunate, where a higher production of gametocytes in patients treated with DHA-PQP was observed [5], [9]. Such an effect has been attributed to the lower dose of artemisinin derivative used in the DHA-PQP. Gametocytaemia is a proxy measure of transmission potential and the increased gametocyte production related to DHA-PQP use may be a public health disadvantage that should be nevertheless balanced against a better PTP, particularly useful in areas of intense transmission.

Dihydroartemisinin-piperaquine was well tolerated, with few adverse events of clinical relevance. A higher frequency of abdominal pain and diarrhoea has previously been reported for DHA-PQP compared with mefloquine-artesunate [8] but this disadvantage of DHA-PQP was not observed in this trial where the comparator treatment was AL. The statistical significant difference in the QTc interval at day 2 was observed only when applying Bazett's but not Fridericia's correction. This was not considered as clinical relevant because of the discrepant results obtained with the 2 methods and because both the proportions of patients with a QTc prolongation between day 0 and 2 higher than 60 ms or with an absolute QTc value greater than 500 ms were extremely low and balanced between groups. Therefore, considering that no cardiovascular AEs were reported, this study adds to the evidence [8] that, at therapeutic doses, DHA-PQP and AL do not have any clinically significant cardiotoxicity.

It has also been previously reported that the only potentially serious adverse effects of artemisinin derivatives are rare type 1 hypersensitivity reactions [8], [32]. However, no evidence of moderate or severe adverse reactions of this kind was observed in the current study and this was despite the larger sample size compared with other published studies in which the number of patients recruited for each arm did not exceed a few hundred. Indeed, it is reassuring that no major safety problem has been observed in more than 1,000 children treated with DHA-PQP. Nevertheless, such a sample size is unable to detect rare and unexpected serious adverse events and the development of a pharmacovigilance system should be a priority, not only for DHA-PQP but also for all other ACTs. African countries should be encouraged, as the use of ACTs increases, to establish pharmacovigilance systems [29] and drug developers and funding agencies should contribute to their development.

In conclusion, DHA-PQP is a safe, efficacious, tolerable and affordable new antimalarial treatment option in Africa. Its longer PTP period may be particularly useful in areas where transmission is intense, though it may exert an important drug pressure on the parasite populations, possibly selecting resistant strains. The deployment of several ACTs as multiple first line treatments may overcome this problem. Indeed, assuming that different treatments are used in equal amounts in the host population, the use of multiple first line therapies would have two main benefits, i.e. the inability of the parasite to adapt to a variable environment and the reduced drug pressure as the rate at which a given treatment is used would be lower than if it was the only one available [33]. DHA-PQP can definitely play an essential role in our effort to reduce the currently high malaria burden.

## Supporting Information

Protocol S1 Trial protocol
(0.46 MB DOC)

Checklist S1 CONSORT checklist
(0.36 MB DOC)
