# Supplementary material for: Dihydroartemisinin-Piperaquine and Artemether-Lumefantrine for Treating Uncomplicated Malaria in African Children: A Randomised, Non-Inferiority Trial
Source: PLoS One. 2009 Nov 17;4(11):e7871. doi: 10.1371/journal.pone.0007871 (PMC2776302; doi:10.1371/journal.pone.0007871)
Supplement: Protocol S1 — Trial protocol (0.46 MB DOC) [file pone.0007871.s001.doc]

**σ sigma-tau**

Industrie Farmaceutiche Riunite S.p.A.

Sede Legale: Roma, V.le Shakespeare 47

Cap. Sociale: 30.500.000.000 inter. versato

Cod. Fiscale: 00410650584

P. IVA n°: 00885531004

Canc. Comm. Trib. di Roma n° 1468/57

Iscriz. Sch. Naz. Ric. Cod. G0120YKG

c.c.i.a.a.: Roma n° 205785


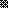


MEDICAL DEPARTMENT - *Research & Development Department*

**Artekin**

Study Protocol No.: ST3073+ST3074 DM040011

(Revised Final Protocol v5.00 incorporating Amendment N° 2D(Mozambique only) dated 8^th^ May 2006

**A Phase III, randomized, non-inferiority trial, to assess the efficacy and safety of Dihydroartemisinin+Piperaquine (DHA+PPQ, Artekin) in comparison with Artemether+Lumefantrine (A+L, Coartem) in children with uncomplicated *P*. *falciparum* malaria.**

**- MULTICENTRE STUDY IN AFRICA-**

**Co-ordinating Investigator**

**Professor Umberto D'Alessandro**

**Prince Leopold Institut of Tropical Medicine**

**Nationalestraat 155**

**B-2000 Antwerp-Belgium**

**Protocol No.:** ST3073+ST3074 DM040011

**Date:** May 8 2006

**Version:** Amendment No 2D (For Mozambique only)

**Title:** A Phase III, randomized, non-inferiority trial, to assess the efficacy and safety of Dihydroartemisinin+Piperaquine (DHA+PPQ, Artekin) in comparison with Arthemeter+Lumefantrine (A+L, Coartem) in children with uncomplicated *P*. *falciparum* malaria.

**Countries :** 5 countries: Mozambique, Kenya, Burkina Faso, Uganda, Zambia.

**Sites :** 5 sites (one site in each country).

**Product:** Dihydroartemisinin+Piperaquine (DHA+PPQ, Artekin™).

**Pharm. Form.:** Tablets containing 20 mg or 40mg of Dihydroartemisinin and 160 or 320 mg of Piperaquine.

**Control Drug:** Artemether+Lumefantrine (A+L, Coartem)

**Pharm. Form.:** Tablets containing 20 mg of Arthemeter and 120 mg of Lumefantrine.

**Sponsor:** Sigma-Tau i.f.r. S.p.A., Pomezia (Rome) – Italy.

**Co-ordinating Investigator:** Professor Umberto D'Alessandro

**Address:** Prince Leopold Institut of Tropical Medicine

Nationalestraat 155, B-2000 Antwerp-Belgium

**Telephone:** 0032 3 247 6354

**Fax:** 0032 3 247 63 59

**STUDY ACKNOWLEDGMENT/CONFIDENTIALITY**

**By signing this protocol, the Investigator(s) acknowledges and agrees:**

The protocol contains all necessary information for conducting study. The Investigator will conduct this study as detailed herein and will make every reasonable effort to complete the study within the time designated.

The protocol and all relevant information on the drug relating to pre-clinical and prior clinical experience, which was provided by the Sponsor, will be made available to all physicians, nurses and other personnel who participate in conducting this study. The Investigator will discuss this material with them to assure that they are fully informed regarding the drug(s) and the conduct of the study.

This document contains information that is privileged or confidential. As such, it may not be disclosed unless specific permission is granted in writing by Sigma-Tau i.f.r. S.p.A., Pomezia (Rome) - Italy or such disclosure is required by federal or other laws or regulations. In any event, persons to whom the information is disclosed must be informed that the information is privileged or confidential and may not be further disclosed by them. These restrictions on disclosure will apply equally to all future information supplied which is indicated as privileged or confidential.

Sigma-Tau i.f.r. S.p.A., Pomezia (Rome) - Italy will have access to any source documents from which Case Report Form information may have been generated. The Case Report Forms and any other data pertinent to this study are the property of Sigma-Tau i.f.r. S.p.A., Pomezia (Rome) - Italy, who may utilise the data in various ways, such as for submission to government regulatory authorities, or in publication of the results of this multicentre study after having obtained the agreement of the principal investigator, who should have the opportunity to comment and agree on these beforehand. All investigators will be included as authors of the publications presenting the overall results of the multicentre study.

The conduct and results of this study will be kept confidential until all sites have completed the study, unless an interim publication or presentation is agreed upon. The results of this multicentre study will be published. Furthermore, each Investigator may independently publish the data at that particular site. The first author on such publication will be defined by the local investigator in agreement with the coordinating investigator. However, to prevent premature disclosure of confidential information, the timing of a separate presentation or publication of the study by the Investigator will be subject to mutual agreement in advance between Sigma-Tau i.f.r. S.p.A., Pomezia (Rome) - Italy and the Investigator. Both parties will have an opportunity to review and comment on any manuscripts or abstracts arising from this study within 3 months of presentation of the first draft.

**Approval Signature Date**

Prof./Dr. …………..…….. _______________________________ ____/____/2005___

Investigator

Prof. Umberto D'Alessandro _______________________________ ____/____/2005___

Co-ordinating Investigator

Dr. Marco Corsi _______________________________ ____/____/2005___

Sigma-Tau - Medical Department

Director

Dr. Antonella Bacchieri _______________________________ ____/____/2005___

Sigma-Tau - Medical Department

Head of Biostatistics and Data Management

Signing this document I declare to have read the paragraph relevant to study acknowledgement and confidentiality and authorise Sigma-Tau i.f.r. S.p.A., Pomezia (Rome) - Italy to record my data on a computerised archive containing all the data pertinent to the study.

**SYNOPSIS**

| Title | **A Phase III, randomized, non-inferiority trial, to assess the efficacy and safety of Dihydroartemisinin+Piperaquine (DHA+PPQ, Artekin) in comparison with Artemether+Lumefantrine (A+L, Coartem) in children with uncomplicated *P*. *falciparum* malaria.** |
| --- | --- |
| Protocol Number | ST3073-ST3074 DM040011 |
| Phase | III |
| Methodology | A phase III, randomized, open label, two arms study. |
| Study Duration | Each patient will be followed for 42 days. |
| Country | 5 countries: Mozambique, Kenya, Burkina Faso, Uganda, Zambia. |
| Study Center(s) | 5 sites (one site in each country). |
| Objectives | Primary objective:  The primary objective of the study is to measure the Day 28, PCR corrected cure rates of Artekin and Coartem and demonstrate that the cure rate of Artekin is non-inferior to that of Coartem (non-inferiority margin = 5%). This cure rate is defined as the proportion of patients with adequate clinical and parasitological response at Day 28 plus those treatment failures identified as new infection by PCR.  In order to compare the study results with the historical ones, the criteria described in the WHO document (“Assessment and Monitoring of Antimalarial Drug Efficacy for the Treatment of Uncomplicated Falciparum Malaria – 2003”. See chapter 7, section 7.3) will be used.  .  Secondary objectives:  - Comparison of the uncorrected Day 28 cure rates of both drugs.  - Comparison of the safety profiles of the two treatments.  - Comparison of times of parasite clearance.  - Comparison of time of fever clearance.  - Comparison of gametocytes (prevalences and densities).  - Comparison of haemoglobin (Hb) changes from day 0 to day 28 and to day 42.  - Comparison of cure rates at D42 (PCR corrected and uncorrected). |
| Number of Subjects | 1500 patients of which 500 are expected to be recruited in Mozambique (1000 DHA+PPQ; 500 A+L) in the modified ITT population (see section 9.1).  . |
| Diagnosis and Main Inclusion Criteria | Males and Females aged between 6 months and 59 months inclusive, body weight at least 5 Kg, microscopically confirmed, monoinfection of *Plasmodium falciparum*, history of fever or presence of fever (axillary temperature at ≥ 37.5 °C), written informed consent. |
| Study Product, Dose, Route, Regimen | Dihydroartemisinin+Piperaquine (DHA+PPQ, Artekin™).  Tablets containing 20 mg or 40 mg of Dihydroartemisinin and 160 mg or 320 mg of Piperaquine. |
| Duration of administration | Three days |
| Reference therapy | Artemether+Lumefantrine (A+L, Coartem).  Tablets containing 20 mg of Artemether and 120 mg of Lumefantrine. |
| Criteria for efficacy evaluation | Primary efficacy endpoint:  The primary efficacy endpoint will be the PCR-corrected adequate clinical and parasitological response (ACPR) at D28.  Secondary efficacy endpoints:  The secondary efficacy endpoints will be:   - Crude or PCR uncorrected adequate clinical and parasitological response (PCR uncorrected ACPR)   The comparison of the uncorrected Day 28 cure rates for both drugs will be performed.   - Fever clearance time (FCT)   Fever clearance time will be defined as the time (in hours) from the start of a patient’s treatment to the first consecutive axillary temperature measurements below 37.5°C.   - Asexual parasite clearance time (PCT)   Asexual parasite clearance time will be defined as the time (in days) from the start of a patient’s treatment to 2 consecutive negative blood slides (collected at different days).   - Gametocyte prevalence will be compared on days 7, 14, 28 and 42. - haemoglobin (Hb) will be measured at day 0, 3 , 28 and 42. - Cure rates at D42 (PCR corrected and PCR uncorrected) |
| Criteria for safety evaluation | Adverse events, laboratory parameters, vital signs, ECG. |
| Statistical Methodology | The primary analysis will be based on a 97.5% (one-sided) Confidence Interval (CI) computed on the difference between the 28-day PCR corrected cure rates of the test and the reference treatments, respectively.  In order to claim that DHA+PPQ is efficacious, the lower limit of the CI must be > -0.05 and the point estimate for the 28-day PCR corrected cure rate of DHA+PPQ must be > 0.90, in both the modified ITT and the Per Protocol populations. |
| First patient in | July, 2005 (expected date) |

**TABLE OF CONTENTS**

[1 BACKGROUND INFORMATION 11](#__RefHeading___Toc98929397)

[1.1 Current Challenges in Malaria Chemotherapy 11](#__RefHeading___Toc98929398)

[1.2 New Antimalarial Therapies 12](#__RefHeading___Toc98929399)

[1.2.1 Chlorproguanil–dapsone (CD) (LapDap) 12](#__RefHeading___Toc98929400)

[1.2.2 Artemether –lumefantrine (AL) (Riamet®, Coartem®) 13](#__RefHeading___Toc98929401)

[1.2.3 Artesunate-amodiaquine and artesunate-sulfadoxine/pyrimethamine 13](#__RefHeading___Toc98929402)

[1.2.4 Artesunate-pyronaridine 13](#__RefHeading___Toc98929403)

[1.2.5 Other drugs in development 14](#__RefHeading___Toc98929404)

[1.2.6 Dihydroartemisinin-piperaquine (DHA+PPQ) (Artekin™) 14](#__RefHeading___Toc98929405)

[1.3 Rationale 14](#__RefHeading___Toc98929406)

[1.4 Study Population 15](#__RefHeading___Toc98929407)

[1.5 Ethical Aspects 15](#__RefHeading___Toc98929408)

[2 TRIAL OBJECTIVES AND PURPOSE 16](#__RefHeading___Toc98929409)

[2.1 Primary Objective 16](#__RefHeading___Toc98929410)

[2.2 Secondary Objectives 16](#__RefHeading___Toc98929411)

[3 TRIAL DESIGN 16](#__RefHeading___Toc98929412)

[3.1 Study Design 16](#__RefHeading___Toc98929413)

[3.2 Primary Endpoint 16](#__RefHeading___Toc98929414)

[3.3 Secondary Endpoints 17](#__RefHeading___Toc98929415)

[3.4 Sample Size 18](#__RefHeading___Toc98929416)

[3.4.1 Sample size computation 18](#__RefHeading___Toc98929417)

[3.4.2 Sample size re-assessment 19](#__RefHeading___Toc98929418)

[3.5 Duration of Patient Follow-up 19](#__RefHeading___Toc98929419)

[3.6 Follow-up Chart 19](#__RefHeading___Toc98929420)

[3.7 Selection of the Patients 22](#__RefHeading___Toc98929421)

[3.7.1 Inclusion criteria 22](#__RefHeading___Toc98929422)

[3.7.2 Exclusion criteria 22](#__RefHeading___Toc98929423)

[3.8 Study Procedures 23](#__RefHeading___Toc98929424)

[3.9 Randomisation 26](#__RefHeading___Toc98929425)

[3.10 Treatment Allocation 26](#__RefHeading___Toc98929426)

[3.11 Study Medications 26](#__RefHeading___Toc98929427)

[3.12 Packaging and Labelling 27](#__RefHeading___Toc98929428)

[3.13 Dosage and Dosing Schedule 27](#__RefHeading___Toc98929429)

[3.14 Drug Dispensing 28](#__RefHeading___Toc98929430)

[4 CONCOMITANT THERAPIES 28](#__RefHeading___Toc98929431)

[4.1 Disallowed Concomitant Drug Therapies 28](#__RefHeading___Toc98929432)

[4.2 Allowed Concomitant Drug Therapies 28](#__RefHeading___Toc98929433)

[4.3 Special Conditions 28](#__RefHeading___Toc98929434)

[4.4 Rescue Treatments 28](#__RefHeading___Toc98929435)

[5 PATIENT WITHDRAWAL CRITERIA 28](#__RefHeading___Toc98929436)

[6 PROTOCOL VIOLATION 29](#__RefHeading___Toc98929437)

[7 SAFETY VARIABLES 29](#__RefHeading___Toc98929438)

[7.1 Adverse Events 29](#__RefHeading___Toc98929439)

[7.1.1 Definition of an adverse event 29](#__RefHeading___Toc98929440)

[7.1.2 Severity, relationship of event to study drug, and outcome 29](#__RefHeading___Toc98929441)

[7.1.3 Defintion of a serious adverse event 30](#__RefHeading___Toc98929442)

[7.1.4 Reporting of adverse events 31](#__RefHeading___Toc98929443)

[7.2 Laboratory Evaluations 31](#__RefHeading___Toc98929444)

[7.3 Vital Signs 32](#__RefHeading___Toc98929445)

[8 CASE REPORT FORM (CRF) 32](#__RefHeading___Toc98929446)

[9 STATISTICS 32](#__RefHeading___Toc98929447)

[9.1 Population Analysed 32](#__RefHeading___Toc98929448)

[9.2 Efficacy 33](#__RefHeading___Toc98929449)

[9.3 Efficacy: secondary analysis 33](#__RefHeading___Toc98929450)

[9.4 Safety and Tolerability 34](#__RefHeading___Toc98929451)

[10 MONITORING AND QUALITY ASSURANCE 34](#__RefHeading___Toc98929452)

[11 DATA MANAGEMENT 34](#__RefHeading___Toc98929453)

[12 INVESTIGATOR RESPONSIBILITY 34](#__RefHeading___Toc98929454)

[13 TRIAL REPORTS 34](#__RefHeading___Toc98929455)

[13.1 Statistical Report 34](#__RefHeading___Toc98929456)

[13.2 Final Clinical Report 35](#__RefHeading___Toc98929457)

[14 ADMINISTRATIVE PROCEDURES 35](#__RefHeading___Toc98929458)

[14.1 Regulatory Authorities and Ethical Review Committee 35](#__RefHeading___Toc98929459)

[14.2 Informed Consent 35](#__RefHeading___Toc98929460)

[14.3 Confidentiality and Publication of Results 35](#__RefHeading___Toc98929461)

[14.4 Protocol Amendments 35](#__RefHeading___Toc98929462)

[15 Study Committees 35](#__RefHeading___Toc98929463)

[15.1 Steering Committee 35](#__RefHeading___Toc98929464)

[15.2 Data Monitoring Board 36](#__RefHeading___Toc98929465)

[16 REFERENCES 37](#__RefHeading___Toc98929466)

[17 APPENDICES 41](#__RefHeading___Toc98929467)

**LIST OF ABBREVIATIONS**

| ACPR : | Adequate Clinical and Parasitological Response |
| --- | --- |
| AE : | Adverse Event |
| ALAT : | ALanine AminoTransferase |
| AMMS : | Academy of Military Medical Sciences |
| AS : | Artesunate |
| ASAT : | ASpartate AminoTransferase |
| BP : | Blood Pressure |
| CI : | Confidence Interval |
| CITIC : | China International Trust and Investment Corporation |
| CPMP : | Committee for Proprietary Medicinal Products |
| CQ : | Chloroquine |
| CRF : | Case Report Form |
| CRO : | Contract Research Organization |
| DBP : | Diastolic Blood Pressure |
| DHA : | Dihydroartemisinin |
| ECG : | Electrocardiogram |
| EDTA : | Ethylenediaminetetraacetic Acid |
| ETF : | Early Treatment Failure |
| FCT : | Fever Clearance Time |
| GCP : | Good Clinical Practice |
| GGT : | Gamma-Glutamyl Transferase (Gamma-Glutamyl Transpeptidase) |
| GLP : | Good Laboratory Practice |
| GMP : | Good Manufacturing Practice |
| h : | Hour |
| Hb : | Haemoglobin |
| HR : | Heart Rate |
| ICH : | International Conference on Harmonization |
| IEC : | Independent Ethics Committee |
| IRB : | Institutional Review Board |
| ITT : | Intention To Treat |
| IU : | International Unit |
| IVR : | Interactive Voice Response |
| KPF : | Kunming Pharmaceutical Factory |
| LCF : | Late Clinical Failure |
| LPF : | Late Parasitological Failure |
| MMV : | Medicines for Malaria Venture |
| MQ : | Mefloquine |
| mg : | Milligram |
| NA : | Not Applicable |
| PCR : | Polymerase Chain Reaction |
| PCT : | Parasite Clearance Time |
| PCV : | Packed Cell Volume |
| PP : | Per Protocol |
| PPQ : | Piperaquine |
| RBC : | Red Blood Cells |
| R&D : | Research and development |
| SAE : | Serious Adverse Event |
| SAS : | Statistical Analysis System |
| SBP : | Systolic Blood Pressure |
| SP : | Sulfadoxine Pyrimethamine |
| TDR : | Tropical Disease Research |
| TF : | Treatment Failure |
| URI : | Upper Respiratory Infection |
| WBC : | White Blood Cells |
| WHO : | World Health Organization |

# BACKGROUND INFORMATION

## Current Challenges in Malaria Chemotherapy

The impact of malaria on the health and economic development of human populations is greatest in the tropics and sub-tropics. Most of the malaria burden is in sub-Saharan Africa where *Plasmodium falciparum* affects particularly young children and pregnant women. One of the main strategies for malaria prevention and control is prompt and effective treatment of cases. However, the number of currently available efficacious antimalarial drugs is small1. In addition, efficacious antimalarials tend to be expensive and more affordable drugs such as chloroquine (CQ), sulfadoxine/pyrimethamine (SP), and amodiaquine (AQ) can no longer be used in many settings due to widespread parasite resistance. In Southeast Asia, mefloquine in combination with artesunate was adopted in the light of increasing resistance to mefloquine monotherapy and has been observed to slow the spread of drug resistance and also reduce malaria transmission. Mefloquine+Artesunate is the recommended first line therapy for uncomplicated *P. falciparum* malaria in Thailand, Cambodia and Myanmar. Artemisinin derivatives are also increasingly used as monotherapy in many endemic countries, although the World Health Organization recommends their use only in combination with another antimalarial drug2. Presently, only few loose (non-fixed)-dose and one fixed-dose artemisinin-based combinations, artemether-lumefantrine are marketed.

Malaria drug resistance is a critical factor that undermines malaria control and hinders the achievement of the Roll Back Malaria (RBM) targets. *Plasmodium falciparum* resistance to CQ and SP is now widespread in most of Sub-Saharan Africa. One factor in the emergence of drug-resistant *P. falciparum* has been the widespread use (often over the counter) of two principal classes of antimalarial drugs; namely, the quinoline (e.g. chloroquine and amodiaquine) and antifolate (sulfadoxine/pyrimethamine) drugs. Indeed a major problem in sub-Saharan Africa has been their continued use for the treatment of malaria even in the face of poor efficacy, because of their affordability and widespread availability. In addition, there is limited access to rapid and microscopy-based diagnosis and efficacious treatment in most African settings. Poverty and the inability to travel to a local clinic has resulted in the widespread practice of self medication at home, often with sub-optimal doses of CQ or SP, with consequent sub-therapeutic drug concentrations in the plasma and potent selective pressure favouring the spread of resistant parasites^3^. Overcoming or slowing the spread of drug resistance requires the adoption of several strategies; one of them being the use of efficacious combination chemotherapy targeting different metabolic pathways, a standard practice for the treatment of viral and bacterial diseases, which is now being adopted for the treatment of uncomplicated *P. falciparum* malaria as well.

The artemisinin derivatives in combination with standard antimalarials are presently being promoted as the best therapeutic option for treating drug-resistant malaria and retarding the development or spread of parasite resistance^3, 4, 5^. The artemisinin derivatives are rapidly-acting antimalarial drugs due to a high intrinsic activity over a relatively broad time window during the erythrocytic development cycle of *P. falciparum* (ter Kuile et al. Exp Parasitol 1993)^6^. Their short elimination half-lives (<1 hour for artesunate) reduces the risk of selecting resistant parasites. Furthermore, they reduce gametocyte carrier rates, density and infectivity. This might explain a reported reduction of transmission on the Thai–Myanmar border^8, 9, 10^. Artemisinin are generally well tolerated and there is an excellent safety record^11^. Data on their safety during pregnancy are limited but encouraging^12^, but they are not recommended during the first trimester, though they can be used in the second and third trimester if better alternatives are unavailable or unsuitable.

Over the last 40-50 years, only a few new antimalarial drugs have been developed. Prior to the Second World War, quinine, pamaquine, chloroquine and mepacrine were developed. These were followed by proguanil and amodiaquine in the 1940s, primaquine and pyrimethamine in the 1950s, S/P in the 1960s, artemisinin in the 1970s, in China and several drugs in the 1980s such as mefloquine, halofantrine and various Chinese compounds – pyronaridine, piperaquine and the artemisinin derivatives (artemether and artesunate). More recently dihydroartemisinin has been developed. However, the use of the Chinese compounds has been confined almost exclusively to China and a few Asian countries. Only recently have the artemisinin derivatives been adopted more widely. In the past decade, three new antimalarial drugs have been registered in Western countries and made available on the open market: atovaquone–proguanil (Malarone; for uncomplicated malaria treatment and prophylaxis in travellers), injectable artemether (Artemotil) for the treatment of severe malaria and artemether–lumefantrine (Riamet/Coartem) for the treatment of uncomplicated malaria.

There is an urgent need for affordable and efficacious antimalarial drugs for the resource-constrained countries in sub-Saharan Africa. The vast majority of malaria cases are treated with cheap but largely ineffective drugs (chloroquine or sulfadoxine/pyrimethamine). The current R&D model is largely based (with few exceptions) on public sector basic research and discovery of products which are then further developed by the private sector. However, the latter model has been ineffective in providing new antimalarial drugs. Only four of approximately 1400 drugs registered worldwide during 1975–1999 were antimalarials^13^. The distribution of Artemisinin and its derivatives is still limited because their development does not meet international criteria. The rules and criteria regulating drug development have changed significantly in the recent past and stricter criteria are currently applied, particularly after the adoption of the International Conference for Harmonisation guidelines. As a consequence, developing a new drug is now more expensive and takes longer. The latter, along with the low solvency of the markets explains in part the low output of new antimalarial drugs by the Western, research-based pharmaceutical industry. The world’s drug market is highly skewed; more than 80% of the US$ 337 billion market in 1999 was in the USA, Europe and Japan, which account for less than 20% of the world’s population. Traditionally, Western countries promoted antimalarial drug research during the colonial era, and in case of war in endemic countries (e.g. mefloquine during the Vietnam war). Today, neither condition exists, and the traveller market alone is not sufficiently attractive/saturated to stimulate the development of new antimalarials. A recent increase in global funding initiatives, however, succeeded in driving the development of a number of new drugs and drug combinations for the treatment of uncomplicated *P. falciparum* malaria. These drugs are in various stages of development.

## New Antimalarial Therapies

The majority of new therapies are combinations of existing antimalarial drugs with an artemisinin derivative.

### Chlorproguanil–dapsone (CD) (LapDap)

This is a fixed-dose antifolate combination that has been developed jointly by GlaxoSmithKline and WHO/TDR (Tropical Disease Research). CD received approval from the UK Regulatory Agency in July 2003, and GlaxoSmithKline and WHO are collaborating on operational research on public health and access issues of CD in resource-poor settings, ahead of its anticipated introduction. The two components in CD have been in use singly for several years, and they were combined in order to obtain an antifolate combination with shorter elimination time than sulfadoxine/pyrimethamine. In addition the combination could lower the probability of selecting resistant parasites. CD compares well with sulfadoxine/pyrimethamine^14^ in controlled studies in East Africa and is effective in patients who have failed on sulfadoxine/pyrimethamine^15^. However, there is some debate regarding its optimal role in treating falciparum malaria in Africa because it shares similar genetic mechanisms of resistance with sulfadoxine/pyrimethamine. Consequently its therapeutic life span might be short. Chlorproguanil–dapsone–artesunate (CDA) is at an early phase of development, with funding from the Medicines for Malaria Venture (MMV), GlaxoSmithKline and WHO/TDR, having completed pre-clinical toxicological studies, and phase II clinical trials are near completion.

### Artemether –lumefantrine (AL) (Riamet®, Coartem®)

This is a fixed-dose combination of artemether (a semi-synthetic artemisinin derivative) and lumefantrine (a slowly eliminated drug also referred to as benflumetol). The registered indications and branding for AL cover treatment of uncomplicated malaria caused by mono or mixed *Plasmodium* infections. The combination is expected to confer mutual protection against resistance and prevent recrudescence after artemether therapy. The components of this combination were originally studied and developed in China by the Academy of Military Medical Sciences (AMMS), Beijing and Kunming Pharmaceutical Factory (KPF), Kunming. The fixed combination has been registered in China since 1992 and has undergone further development when Novartis signed a collaborative agreement in 1994 with AMMS, KPF and CITITEC, the technology arm of the China International Trust and Investment Corporation (CITIC). Studies for the international registration started in 1995. AL was registered in Switzerland in 1999 and has since received marketing authorisation in several endemic and non-endemic countries. Coartemether is marketed under a dual-branding, dual-pricing strategy. Riamet® (six doses over either 3·days or 5·days) is available in developed, non-endemic countries for treatment of malaria among travellers at a price comparable with the latest antimalarial introductions. Coartem® is registered and marketed in malaria-endemic countries as either a four (no longer recommended) - or six-dose treatment of uncomplicated falciparum malaria at prices comparable with locally available products. Recently, an agreement was reached between Novartis and WHO for AL to be made available to the public sector of developing countries at a preferential price. Few clinical trials, mostly with the four-dose regimen, have been conducted both with the original Chinese combination product and the subsequent product used for international registration. A Cochrane review ^16^ has, in 2003, identified eight randomized, controlled trials comparing coartemether with standard treatment for uncomplicated falciparum malaria (2117 participants). From the meta-analysis, it was concluded that the four-dose coartemether regimen was superior to chloroquine and equivalent to S/P in areas of chloroquine resistance but inferior to mefloquine and mefloquine-artesunate in areas of multidrug resistance. The six-dose regimen was also equivalent to mefloquine-artesunate but was better tolerated ^17, 18^. A collaborative project between Novartis and WHO has led to a more user-friendly packaging of the six-dose treatment, which is now being field tested. A paediatric formulation is also being developed and trials have been conducted for regulatory submission to extend the label to cover children who weigh less than 10 kg.

### Artesunate-amodiaquine and artesunate-sulfadoxine/pyrimethamine

Several clinical trials involving the latter drugs have been completed in Africa, while Artesunate-mefloquine has been widely used in Thailand and other parts of Southeast Asia as a loose formulation, and has been proven to be highly efficacious in areas of mefloquine resistance. Artesunate-amodiaquine and artesunate-mefloquine will be further developed as fixed-dose combinations by WHO/TDR (Drugs for Neglected Diseases/populations) and will undergo all the relevant pre-clinical and clinical studies before registration. In addition, a blister pack of artesunate-sulfadoxine/pyrimethamine and artesunate-amodiaquine have been developed using age-based dosing and is being used in clinical trials in Africa.

### Artesunate-pyronaridine

Artesunate-pyronaridine builds on the rationale of using an artemisinin derivative with a longer-acting partner drug. **Pyronaridine** is an antimalarial compound that has been synthesized and developed in China, where it obtained marketing authorization for the treatment of malaria in the 1980s. It has proven efficacy against drug-resistant falciparum malaria in Africa^19^. The efficacy in Thailand of the polybioavailable formulation against multi drug-resistant malaria was less than 90% ^20^. In the same setting, a better formulation was >95% efficacious^21^.

### Other drugs in development

**Artemisone** is a metabolically stable semi-synthetic derivative of artemisinin that is being developed by Bayer. Several trioxanes obtained by total synthesis are now available and are being assessed for further development by MMV. **Isoquine** is an isomeric derivative of amodiaquine that might not generate the toxic quinone-imine metabolites that are thought to have a role in the development of neutropenia and hepatitis. The development of the latter drug is also being coordinated by MMV. Short-chain chloroquine analogues with better efficacy on chloroquine-resistant isolates are being researched at Tulane University. A novel compound, **fosmidomycin**, has recently been tested in small numbers of patients. Fosmidomycin inhibits the 1-deoxy-D-xylulose 5-phosphate reductoisomerase, an enzyme of the nonmevalonate pathway of isoprenoid biosynthesis, which is absent in humans but present in apicomplexan parasites (Jomaa, Science 1999). Fosmidomycin was well tolerated and acted rapidly, but late asexual parasite recrudescences with treatment durations of less than 4 days preclude its use as monotherapy^22, 23^. The phase II clinical development of fosmidomycin combinations is at advanced stage and phase III studies are currently planned (Borrmann et al. 2004).

### Dihydroartemisinin-piperaquine (DHA+PPQ) (Artekin™)

Artekin™ was developed in China and is registered in China and Cambodia. Non GMP DHA-PPQ has been evaluated extensively in clinical trials in Thailand, Vietnam, Cambodia and China^24^, efficacy has been high and tolerability uniformly good in all trials in multidrug-resistant areas, where piperaquine-resistance was common after its extensive use for mass prophylaxis. Initially, the co-formulation included primaquine and trimethoprim (CV8), which is still part of national policy in Vietnam. Piperaquine is an orally active bisquinoline discovered by Rhône-Poulenc in the early 1960s and developed for clinical use in China in 1973. Piperaquine is approximately equivalent to chloroquine against sensitive parasites, but is significantly more effective than chloroquine against resistant *P. falciparum.* Piperaquine replaced chloroquine as the recommended treatment for falciparum malaria in China in 1978. Overall, 194 140·kg of piperaquine phosphate, equivalent to 140 000·000 adult doses, were used for mass prophylaxis and treatment. Surveillance at the time found no adverse events other than rare cases of a rash. However, reports about emerging parasite resistance to piperaquine from southern China, an area of intensive use of piperaquine, are of concern. Dihydroartemisinin is the active metabolite of artesunate and artemether. It has equivalent clinical efficacy to the more widely used artesunate. A development programme has been agreed between Holleykin Pharmaceuticals and Guangzhou University (China), The University of Oxford, MMV, and Sigma-Tau Industrie Farmaceutiche Riunite SpA to support the international registration of the drug.

## Rationale

The fight against malaria currently relies on early curative treatment of malaria, particularly in children, in order to decrease mortality and morbidity ^31^. Recent assessments confirm the persistence of endemic malaria with an estimated 400 million cases and 1.5–2.7 million deaths per year. In sub-Saharan Africa alone, *Plasmodium falciparum* is responsible for approximately 220 million cases and one million deaths per year, 75% of them being children under 5 years old.

The drug resistance of *P. falciparum* to CQ was first reported in the 1960s. Resistance to SP was reported later in the 1980s, and has now spread globally. After several centuries of use, natural quinine is still effective against *P. falciparum* everywhere in the world except in Southeast Asia and South America, where decreased susceptibility is reported. No clinical resistance to the artemisinin derivatives has been observed, despite their use for 15 years in Asia and, more recently, in Africa. Nonetheless, the current state of parasite resistance to other antimalarial drugs worldwide is worrying, hence the need to develop new therapeutic options for the treatment of *P. falciparum* malaria by simultaneously reassessing old and forgotten drugs, while searching for new drugs, and identifying synergistic drug combinations.

The elimination half-life of antimalarial drugs is an important factor with respect to erythrocytic schizonticidal action (the killing of parasitized red cells with asexual forms) because sufficiently high drug concentrations must be maintained for at least three life cycles, i.e., 6 days. Therefore, treatment with drugs that have a short half-life (less than 1 day ) must be continued for 6–7 days if given as monotherapy. Compounds with a long half-life, i.e., greater than 5 days, have the advantage of allowing treatment for 1–3 days. Conversely, however, sub-inhibitory plasma concentrations associated with antimalarial drugs with a long terminal elimination half-life might contribute to the selection and spread of resistant alleles.

Presently, there are two philosophies that guide the selection of drug combinations: 1) combining two drugs with similar and preferably short (chlorproguanil and dapsone) or intermediate (sulfadoxine and pyrimethamine) half-lives; 2) combining two drugs with different but complementary, half-lives; one short and the other long, which leads to a risk of de facto monotherapy when the shorter half life product is eliminated, a risk that could be minimized by choosing a compound that is rapidly effective on 95% of the parasite population such as the artemisinin derivatives. The partner drug with the longer half-life such as mefloquine, lumefantrine, amodiaquine or piperaquine has a smaller parasite biomass to clear and hence a low risk for the selection of resistant parasites during the initial treatment phase.

Moreover, compliance is another important factor to take into consideration when developing a new combination. DHA+PPQ is considered a highly efficacious and safe combination that is likely to have an optimal risk/benefit ratio in malaria therapy. Artekin™ is a second generation Artemisinin-based Combination Therapy (ACT), with similar efficacy to that of Coartem (artemether+lumefantrine) or Artesunate+Mefloquine, but with a simpler dosing scheme that will aid better compliance. Moreover, its apparent good safety profile and affordable cost make it ideal for resource constrained countries. Most of the clinical trials evaluating Artekin™ have been conducted without the sponsorship of a pharmaceutical company. In addition, the formulation of DHA+PPQ used in some trials^24, 25, 26, 27, 28, 29^ , though compliant with Chinese GMPs, was not compliant with the GMP standards required by the European or US Health Authorities. For the latter reasons Sigma-Tau has used the information/data generated from previous trials to support the design of a new Phase III development strategy using Artekin™ produced according to good GMP standards.

## Study Population

Patients selected for this trial will be paediatric outpatients of both sexes, affected by uncomplicated *P falciparum* malaria.

## Ethical Aspects

Prior to commencement of the studies, the trial protocol shall be submitted to the relevant Independent Ethical Committee (IEC) or Institutional Review Board (IRB) for approval. The EEC/IRB’s written approval of the study shall be appended to the standard study documents at the sponsor and at the location of the investigator(Trial Master File; TMF). All patients will receive a full explanation of the proposed study with the potential risks and benefits from the Investigator or one of the medical research personnel specifically designated by the Investigator. Prior to entering the study, written informed consent will be obtained and a copy of it will be provided to the patient (see Appendix IV). Patients will be free to withdraw from the study at any time for any reason and their decision to discontinue participation will in no way jeopardise their access to prompt health care at the Investigational Centre. The Investigator declares by signing this protocol that he/she will perform the study in compliance with the protocol, scrupulously following the Good Clinical Practice (GCP) and all the applicable regulatory requirements.

# TRIAL OBJECTIVES AND PURPOSE

The aim of the study is to determine whether a new formulation of DHA+PPQ is at least as good as (not inferior to) A+L for the treatment of African children with acute uncomplicated *P. falciparum* malaria and to assess its safety and tolerability.

## Primary Objective

The primary objective of the study is to measure the Day 28, PCR corrected cure rates of Artekin and Coartem and demonstrate that the cure rate of the former treatment is non-inferior to that of the latter (non-inferiority margin = 5%). This cure rate is defined as the proportion of patients with adequate clinical and parasitological response at Day 28 plus those treatment failures identified as new infection by PCR.

In order to compare the study results with the historical ones, the criteria described in the WHO document (“Assessment and Monitoring of Antimalarial Drug Efficacy for the Treatment of Uncomplicated Falciparum Malaria – 2003”. See chapter 7, section 7.3) will be used, i.e. the failure rates computed in each treatment group individually considered will be judged against the efficacy threshold of 90%.

## Secondary Objectives

The secondary objectives of the study will be the between treatment comparison of:

- the uncorrected Day 28 cure rates of both drugs (risk for rescue therapy);

- the safety profiles of the two treatments;

- times of parasite clearance (PCT);

- time of fever clearance (FCT);

- gametocyte prevalences and densities on days 7, 14, 28, 42;

- haematological recovery (Haemoglobin (Hb) changes from day 0 to day 28 and day 42;

- cure rates at D42 (PCR corrected and PCR uncorrected).

# TRIAL DESIGN

## Study Design

This is a phase III, randomized, open label, two-armed study involving 5 investigational centres which will enroll 1,500 patients (1,000 DHA+PPQ; 500 A+L).

The main justification for not performing a double-blind study is the requirement to use the double-dummy technique requiring placebos for DHA+PPQ and for A+L, resulting into a treatment schedule which would be very laborious for the patient. In order to ensure concealment of treatment allocation and avoid other biases, the following requirements will be assured:

1. the randomization list will be generated prior the beginning of the study. Treatment allocation will be concealed until the completion of the screening and the final recruitement of the patient (see section 3.9);
2. the interpretation of the PCR reading will be blinded/masked with regard to the treatment allocation of the patients (see section 3.8); for quality control an independent laboratory will carry out PCR analysis on a percentage of blood samples;
3. an independent Data Monitoring Board will review a significant percentage of efficacy data and all safety data (see section 15.2).

## Primary Endpoint

The primary endpoint will be the PCR-corrected adequate clinical and parasitological response (PCR corrected ACPR) at D28.

ACPR is defined as the absence of parasitaemia on D28 irrespective of the axillary temperature without previously meeting any of the criteria of early treatment failure or late clinical or parasitological failure.

Patients with late asexual parasite reappearance will be considered ACPR if the PCR analysis shows a new infection rather than a recrudescence.

The total treatment failure is defined according to the WHO criteria (WHO 2003) as the sum of early* and late** treatment failures.

* Early Treatment Failure (ETF)

*(i) Development of danger signs or severe malaria on Day 0, Day 1, Day 2 or Day 3, in the presence of parasitaemia,*

*(ii) Parasite density on Day 2 > Day 0 count irrespective of axillary temperature,*

*(iii) Presence of parasitaemia on Day 3 with fever (axillary temperature ≥ 37.5°C),*

*(iv) Parasitaemia on Day 3 ≥ 25 % of count on Day 0.*

** Late treatment failure (LTF)

Late Clinical Failure (LCF)*:*

*(i) Development of danger signs or severe malaria after Day 3 in the presence of parasitaemia, (See Appendix V for the critera for severe malaria/danger signs)*

*(ii) Presence of parasitaemia and fever on any day from Day 4 to Day 28, without previously meeting the criteria of ETF.*

Late Parasitological Failure (LPF)*:*

*Reappearance of parasitaemia after initial clearance between day 4 and day 28 (identified as recrudescent infection by PCR analysis) in the absence of fever (axillary temperature <37.5°C) without previously meeting the criteria of ETF or LCF.*

## Secondary Endpoints

The secondary endpoints will be:

- Crude (PCR uncorrected) adequate clinical and parasitological response (PCR uncorrected ACPR)
- Fever clearance time (FCT)

Fever clearance time will be defined as the time (in hours) from the start of a patient’s treatment to the first consecutive axillary temperature measurements below 37.5°C (body temperature <37.5ºC for at least 48 hours).

- Asexual parasite clearance time (PCT)

Asexual parasite clearance time will be defined as the time (in days) from the start of a patient’s treatment to 2 consecutive negative blood slides (collected at different days).

- Gametocyte carrier rates and geometric mean densities (excluding negative slides) will be compared on days 7, 14, 28 and 42.
- Haemoglobin (Hb) will be measured at day 0, 3, 28 and 42.

,

- Cure rates at D42 (PCR corrected and PCR uncorrected)
- Safety profiles of the two treatments

Adverse events, vital signs, blood chemistry and haematology, electrocardiogram (ECG) will be monitored and changes in relevant laboratory parameters will be assessed.

## Sample Size

3.4.1 Sample size computation

The study is designed as a non-inferiority trial.

The level of the PCR-corrected cure rate at day 28 for Arthemeter+Lumefantrine has been estimated, through a literature search, to be in the range 90%-92%^32-33^ in the Intention-To-Treat (ITT) population. The opinion of physicians and experts in malaria treatment has been sought for defining the non-inferiority margin, and this has been set at 5%.

An unequal randomization with 2:1 structure was chosen because it assures a larger sample size for the DHA+PPQ group which provides the following advantages:

- a more precise estimate of the DHA+PPQ cure rate;
- a bigger sample for the integrated safety data base of DHA+PPQ, that renders more likely the elucidation of rare adverse reactions, if any.

From an ethical point of view, this choice appears reasonable in view of the previous data on DHA+PPQ, in terms of both safety and efficacy.

Sample size was initially (see original version of the protocol) computed considering the original formulation of the primary objective, which required the use of a simulation (details are provided in a separate document - see Sample Size and Design Justification for The Pivotal Clinical Trials of Dihydroartemisinin+ Piperaquine**)**.

Having divided the two criteria of non-inferiority and comparison of study results with historical ones, power has been recomputed for each of the two objectives as described below**.**

For the non-inferiority analysis, the clinical and statistical specifications for the sample size computations are as follows:

- Primary endpoint = PCR-corrected cure rate at day 28.
- Primary analysis = based on 97.5% one-sided confidence intervals for the difference in cure rates.
- Alpha = 0.025 (one-sided).
- Power = 80%.
- Cure rate of Coartem in the pure ITT population = at least 91%.
- Non-inferiority margin for the difference (test-reference) = -0.05.
- Randomization: based on a 2 :1 allocation scheme (test : reference).
- Rate of patient attrition in the modified intention-to-treat population as compared to the pure ITT population (i.e. rate of non-informative withdrawals) = 10%. Expected 28-day PCR corrected cure rate in this population = 91%.
- Rate of patient attrition in the Per Protocol Population as compared to the ITT population (i.e. rate of withdrawals for any reason and protocol violations) = 30%. Expected 28-day PCR corrected cure rate in this population = 93%.
- Primary Analysis Populations = both the modified Intention To Treat (ITT) and the Per Protocol Populations.

With these assumptions, the sample size of 1500 patients (1000 in the Artekin group and 500 in the Coartem group) provides a power of approximately 83% for the lower bound of a 97.5 one-sided confidence interval for the treatment difference being above –0.05 in the modified ITT and 82% for the same analysis in the Per Protocol population (for the power calculation both continuity correction and inequality of variances under the null hypotheses have been considered).

For the historical comparisons, the clinical and statistical specifications for the sample size computations are as follows:

- Anticipated population proportion of clinical failures = as high as 10%.
- Confidence level = 95% (two-sided interval).
- Precision = 5 percentage points.
- Rate of patient attrition in the Per Protocol Population as compared to the ITT population (i.e. rate of withdrawals for any reason and protocol violations) = 30%.

According to Annex 4 of the WHO document “Assessment and Monitoring of Antimalarial Drug Efficacy for the Treatment of Uncomplicated Falciparum Malaria – 2003”, a sample size of at least 200 patients would be required in each treatment group for estimating the proportion of clinical failures to within a 5 percentage point of the true value with 95% confidence.

### 3.4.2 Sample size re-assessment

Due to the limitation of available data on the cure rate of Arthemeter+Lumefantrine (six doses) in the target population and to the uncertainty regarding the expected rate of withdrawals, an interim analysis will be performed solely for sample size re-assessment. This analysis will be carried-out when half of the patients originally planned (i.e. 750 patients) are randomized and have reached the Day 28 evaluation.

This analysis will be performed for checking:

- the overall rate of patients withdrawing from the study considering all the possible reasons;
- the proportion of non evaluable PCR;
- the overall 28-day PCR corrected cure rate.

These evaluations will be carried-out in each of the two primary populations (i.e. modified ITT and PP populations) by the Data Monitoring Board. The sample size will be recomputed based on these findings and changed accordingly, if an increase is needed (i.e. no downsizing will be implemented). As a general rule, the Data Monitoring Board will base the above specified evaluations on blind data.

The sample size resulting from this assessment (either if necessitating an adjustment or not) will not be communicated to the Investigators so as not to reveal any information on the primary outcome (rate of cure) of the study.

The method for performing this analysis could be based on the papers by A.L. Gould^34^ and J Herson and J. Wittes^35^. However, such methods apply to superiority studies and therefore need to be adapted for the non-inferiority case. All details of this interim analysis will be provided in the Statistical Analysis Plan (or a separate document) that will be finalized before starting any interim analysis.

## Duration of Patient Follow-up

Each patient will be followed for 42 days.

## Follow-up Chart

All children attending the outpatient clinic with fever or history of fever in the preceding 24 hours will have a thick and thin blood smear done. The parents or guardians of those with a *P. falciparum* infection of the required density will be informed by the health facility staff about the malaria clinical trial and will be requested to freely consent to participate in the trial. Unwillingness or inability to participate will result in the prescription and administration of the local standard treatment regime for uncomplicated *P. falciparum* malaria. The parents or guardians who accept their child to participate in the study will receive detailed explanations about the trial from the study staff. Specifically they will be informed that two antimalaria drugs are being tested and the option given to the child will be decided randomly (by chance). They will be asked to sign the informed consent form (see Appendix IV).

For children who meet the enrollment criteria, the following steps will be taken:

1. A Case Report Form consisting of demographic and physical/clinical information will be completed;
2. Body weight and axillary temperature will be measured (the latter by using a digital electronic thermometer) and the results will be recorded on the Case Report Form. If the initial temperature value is less than 36°C the measurement will be repeated.
3. A thick blood smear will be obtained for measurement of parasitatemia.
4. A blood sample will be collected on Whatman filter paper number 3MM for subsequent genotyping of the infective parasite strains.

At enrolment, children will be assigned a sequential study number. During the first 3 study days (days 0-2) patients will be either admitted for observation and study drug administration or managed as outpatients requiring home visits for supervised study drug administration at night time. Parents/guardians will then be encouraged to return to the clinic for follow up assessments on days 3, 7, 14, 21, 28, 35, 42 and on any unscheduled day if the child is not well.

Compensation for transportation to and from the clinic will be provided.

All medication will be administered under observation by an authorised member of the study team (physician, clinician, or study nurse).

To decrease the risk of new infections all children who have been screened for the study whether or not they agree to participate, will receive an insecticide-treated bed net (long-lasting) at recruitment. The mother/guardian will be asked to let the child sleep under the bed net. At the end of the follow up, the mother/guardian will receive an additional insecticide-treated bed net.

Patients will be assessed as summarized in the following flow-chart.

Figure 1: Follow-up chart

|  | **Day 0** | **Day 1** | **Day 2** | **Day 3** | **Day 7** | **Day 14** | **Day 21** | **Day 28** | **Day 35** | **D42** | **Day of any recurrent parasitemia** |
| --- | --- | --- | --- | --- | --- | --- | --- | --- | --- | --- | --- |
| ***Visits*** | ***V1*** | ***V2*** | ***V3*** | ***V4*** | ***V5*** | ***V6*** | ***V7*** | ***V8*** | ***V9*** | ***V10*** |  |
| **Demographic data/Medical history** | **x** |  |  |  |  |  |  |  |  |  |  |
| **Informed consent recording** | **x** |  |  |  |  |  |  |  |  |  |  |
| **Physical/clinical examination** | **x** | **x** | **x** | **x** | **x** | **x** | **x** | **x** | **x** | **x** | **x** |
| **Vital signs and weight^1^** | **x^1^** | **x** | **x** | **x** | **x** | **x** | **x** | **x** | **x** | **x** | **x** |
| **Blood smear** | **x** | **x** | **x** | **x** | **x** | **x** | **x** | **x** | **x** | **x** | **x** |
| **Electrocardiogram (ECG)** | **x** |  | **x** |  | **x** |  |  | **X^4^** |  | **X^5^** | **x** |
| **Hematology** | **x** |  |  | **x** | **x^2^** | **x^3^** |  | **x** |  | **x** | **x** |
| **Clinical chemistry** | **x** |  |  | **x** | **x^2^** | **x^3^** |  | **x** |  | **x** |  |
| **Adverse events recording** | **x** | **x** | **x** | **x** | **x** | **x** | **x** | **x** | **x** | **x** | **x** |
| **PCR sampling** | **x** |  |  |  |  | **x** | **x** | **x** | **x** | **x** | **x** |
| **Concomitant treatments Recording** | **x** | **x** | **x** | **x** | **x** | **x** | **x** | **x** | **x** | **x** | **x** |
| **Study medications** | **x** | **x** | **x** |  |  |  |  |  |  |  |  |

*1. Weight will be measured at D0*

*2. If abnormal at D3*

*3. If abnormal at D7*

*4. Only if abnormal at D7*

*5.Only if abnormal at D28*

## Selection of the Patients

### Inclusion criteria

In order to be eligible, patients should satisfy the following inclusion criteria:

1. Males and Females aged between 6 months and 59 months inclusive.
2. Body weight of 5 Kg and above.
3. Microscopically confirmed, monoinfection of *Plasmodium falciparum* (parasitaemia ≥ 2000/μL to 200,000/μL).
4. History of fever in the previous 24 hours or presence of fever (axillary temperature at ≥ 37.5°C).
5. Signed informed consent by the parents or guardians.

6. Parents’ or guardians’ willingness and ability to comply with the study protocol for the duration of the trial.

### Exclusion criteria

Patients with any of the following criteria will not be admitted to the study:

1. Participation in any investigational drug study during the previous 30 days.

2. Known hypersensitivity to the study drugs.

1. Severe malaria.
2. Danger signs: not able to drink or breast-feed, vomiting (> twice in 24hours), recent history of convulsions (>1 in 24h), unconscious state, unable to sit or stand.
3. ECG abnormality that requires urgent management.
4. Presence of intercurrent illness or any condition which in the judgement of the investigator would place the subject at undue risk or interfere with the results of the study.
5. Severe malnutrition (defined as weight for height <70% of the median NCHS/WHO reference.
6. Ongoing prophylaxis with drugs having antimalarial activity such as cotrimoxazole for the prevention of Pneumocisti carini pneumonia in children born to HIV+ women.

## Study Procedures

The critical steps for the study period are described in Appendix VIII.

**Visit 1 (Day 0): screening visit/ administration of the study medication**

1. Informed Consent

A signed informed consent from the parent/guardian shall be obtained before any tests or evaluations related to the study eligibility are carried out.

2. Demographic Data and Medical History

Demographic data and a general history of past and present illnesses will be recorded.

3. Physical and Clinical Examination

A general physical examination will be performed (see Appendix I).

A clinical examination will be performed (see Appendix I): symptoms, axillary temperature (electronic thermometer).

3. Vital Signs and Weight

Vital signs (systolic and diastolic blood pressure, heart rate) and weight will also be measured.

4. PCR

A blood sample will be collected on filter paper (Whatmann 3MM) at Day 0 before treatment and every time a blood slide is done starting at and including D 14. Samples from patients classified as late treatment failure will be subsequently used for parasite genotyping.

5. Blood Slide

A thick and thin blood smear will be obtained from the subject to verify the presence of *P. falciparum* and to calculate the parasite density. Thick and thin blood films will be prepared, dried and stained with Giemsa stain according to standard operating procedures.

Parasite density will be calculated by counting the number of asexual parasites per 200 leukocytes in the thick blood film, based on a assumed WBC of 8,000 /µl by light microscopy at 1000xmagnification. One hundred high-powered fields (HPF) will be examined (independent of presence or absence of asexual parasite stages). The parasite density per microlitre will be calculated using the following formula:

Parasite density / µl = Number of parasites counted x 8,000

Number of leukocytes counted

6. Laboratory Tests

Blood haemoglobin, red blood cell count, total white blood cell count, differential count (neutrophils, basophils, eosinophils, monocytes, lymphocytes) and platelet count.

Total bilirubin, ALAT and creatinine will be measured.

7. Electrocardiogram (ECG)

A 12 lead ECG will be performed and QT/QTc interval prolongation will be evaluated. **Any patient found to have an abnormal ECG throughout the course of the study will be treated in accordance with local practice and national guidelines.**

8. Administration of the Study Drugs

**Visit 2 (Day 1): *Open Label Treatment Period***

1. Physical and Clinical Examination

A general physical examination and a clinical examination will be performed: symptoms, axillary temperature (electronic thermometer).

2. Vital Signs

Vital signs (systolic and diastolic blood pressure, heart rate) will be measured.

3. Blood Slide

A thick and thin blood smear will be obtained to verify the presence and to determine the density of asexual and sexual stages of *P*. *falciparum*.

4. Concomitant Pharmacological Treatments

Concomitant medications being taken by the patients will be recorded. For a list of allowed and disallowed medications, see section 4.

5. Adverse Events Report

The start and end of clinical adverse events will be recorded.

6. Administration of the Study Drugs

**Visit 3 (Day 2): *Open Label Treatment Period***

1. Physical and Clinical Examination

A general physical examination and a clinical examination will be performed: symptoms, axillary temperature (electronic thermometer).

2. Vital Signs

Vital signs (systolic and diastolic blood pressure, heart rate) will be measured.

3. Blood Slide

A thick and thin blood smear will be obtained to determine the presence and the density of asexual and sexual stages of *P*. *falciparum*.

4. Concomitant Pharmacological Treatments

Concomitant medications being taken by the patients will be recorded. For a list of allowed and disallowed medications, see section 4.

5. ECG

A 12 lead ECG will be performed.

6. Adverse Events Report

The start and end of clinical adverse events will be recorded.

7. Administration of the Study Drugs

**Visit 4 (Day 3): *Open Label Treatment Period***

1. Physical and Clinical Examination

A general physical examination and a clinical examination will be performed: symptoms, axillary temperature (electronic thermometer).

2. Vital Signs

Vital signs (Systolic and Diastolic Blood Pressure, Heart Rate) will be collected.

3. Blood Slide

A thick and thin blood smear will be obtained to determine the presence and the density of asexual and sexual stages of *P*. *falciparum.*

4. Concomitant Pharmacological Treatments

Concomitant medications being taken by the patients will be recorded. For a list of allowed and disallowed medications, see section 4.

5. Adverse Events Report

The start and end of clinical adverse events will be recorded.

6. Laboratory Tests

Blood haemoglobin, red blood cell count, total white blood cell count, differential count (neutrophils, basophils, eosinophils, monocytes, lymphocytes) and platelet count.

Total bilirubin, ALAT and creatinine will be measured.

**Visits 5, 6, 7 (Days 7, 14, 21)): *Open Label Treatment Period***

During these visits, the same procedures as the visit 4 will be applied. For Visit 5 (D7), ECG will also be performed..Starting from Day 14, filter paper for PCR analysis.

Laboratory tests will be performed at Day 7 and D14 in case of abnormality at D3 and D7, respectively.

**Visit 8 (Day 28): *Open Label Treatment Period***

1. Physical and Clinical Examination

A general physical examination and a clinical examination will be performed: symptoms, axillary temperature (electronic thermometer).

2. Vital Signs

Vital signs (Systolic and Diastolic Blood Pressure, Heart Rate) will be collected.

3. Blood Slide

A thick and thin blood smear will be obtained to determine the presence and the density of asexual and sexual stages of *P*. *falciparum.*

Filter paper for PCR analysis.

4. Laboratory Tests

Blood haemoglobin, red blood cell count, total white blood cell count, differential count and platelet count.

Total bilirubin, ALAT and creatinine will be measured.

The Investigator(s) will check all laboratory tests results and report them onto the CRF and will indicate those with potential clinical relevance. An abnormal laboratory test value of potential clinical relevance will be reported using the Adverse Event Form attached to the CRF.

5. Concomitant Treatments

Concomitant medications being taken by the patients will be recorded. For a list of allowed and disallowed medications, see section 4.

6. ECG

A 12 lead ECG will be performed.

QT/QTc interval prolongation will be evaluated. only if result of the ECG performed on day 7 was found to be abnormal.

7. Adverse Events Report

The start and end of clinical adverse events will be recorded.

**Visit 9 (Day 35): *Follow-up Period***

During this visit, the same procedures as the visit 7 will be applied.

**Visit 10 (Day 42): *Follow-up Period***

During this visit, the same procedures as the visit 8 will be applied. (ECG evaluation **only if the result of the ECG performed on day 28 was found to be abnormal.**

).

**Unscheduled visits throughout follow up**

During this visits, the same procedures as visits 5, 6, 7 will be applied. Hb will be measured if the patient is classified as treatment failure.

The PCR readings will be masked to the treatment allocation of study subjects, i.e. by personnel different from the treating physician/investigator. In addition, a centralized and independent double-check of a significant percentage of parasitological slides and filter paper blood samples (PCR) will be carried-out. The percentages of slides and films to be reviewed and the corresponding statistical justifications will be specified in the Statistical Analyis Plan.

## Randomisation

Patients will be randomly assigned to one of the two treatment groups (Artekin or Coartem). Blinding in the randomization process will be attained by the use of sealed envelopes. A procedure for guiding to the correct use of the sealed envelopes will be put in place.

Complete blocks of treatment materials will be sent to the investigation centers.

The randomization list will be generated by MDS Pharma Services, using the plan procedure of SAS.

An unequal randomization, 2:1 (Test:Reference), will be used to provide more precise estimates of the DHA+PPQ cure rates, as well as to provide more patients for the integrated safety data base of the combination therapy under evaluation.

## Treatment Allocation

Treatment allocation and administration of medications will be performed by the study nurse or by the investigator at the clinic.

## Study Medications

The clinical trial material (CTM) to be evaluated in this study will be prepared and provided by Sigma-Tau i.f.r. S.p.A., Pomezia (Rome), Italy.

***Dihydroartemisinin (DHA) and Piperaquine (PPQ) = Artekin (Sigma-Tau)***

DHA-PPQ will be co-formulated as fixed-dose film tablets containing 20 mg or 40 mg of dihydroartemisinin and 160 mg or 320 mg of piperaquine.

***Artemether (A) and Lumefantrine (L) = Coartem (Novartis)***

AL will be co-formulated as fixed-dose film tablets containing 20 mg of artemether and 120 mg of lumefantrine.

The stability of the products used in the study will be determined in advance to assure suitability for use throughout the study. The results of this analyses will be included in the Trial Master File.

The CTM shall be stored in a secure area (e.g. pharmacy) until use.

## Packaging and Labelling

Each centre will receive a documented number of packages containing tablets of study drugs for the treatment of one subject (plus extra tablets for potential redosing, maximum 2 redosings). Each package will be identified by a label indicating at least the following information:

- Product name
- Trial reference
- Name and address of the Sponsor
- Batch number and expiry date
- Storage directions
- For clinical Trial only

## Dosage and Dosing Schedule

***Dihydroartemisinin (DHA, 20/40 mg) and Piperaquine (PPQ, 160/320 mg) = Artekin (Sigma-Tau)***

Medications will be administered as close as possible at the following times:

Day 0: hour “X”

Day 1: hour “X” + 24 hours

Day 2: hour “X” + 48 hours

The number of tablets will be based on bodyweight (see the relevant table in Appendix II).

***Artemether (A, 20 mg)) and Lumefantrine (L, 120 mg) = Coartem (Novartis)***

Medications will be administered at the following times:

Day 0: hour “X”

Day 0: hour “X” + 8 hours

Day 1: hour “X” + 24 hours

Day 1: hour “X” + 36 hours

Day 2: hour “X” + 48 hours

Day 2: hour “X” + 60 hours

The drug will be given at specific hours according to the manufacturer of Coartem. A glass of milk will be given to the child immediately after each administration of Coartem.

The number of tablets will be based on bodyweight.

## Drug Dispensing

The study medication will be administered at the clinic or at home by authorized members of the study team. Study medications given to infants and young children (<2 years old) will be crushed, mixed with water, and administered as a slurry if they are unable to swallow. Study medications administered to older children will be given as tablets or fractions of tablets to be taken orally with a glass of water. The study nurse or the investigator will directly observe the intake of study medications. Patients will be observed for one hour to ensure that the medications are not vomited or spat out. Any patient who vomits or otherwise expels the medication within 30 minutes of administration will be retreated with a second dose. If vomiting occurs between 30 and 60 minutes after treatment, only half of the original dose will be given.

# CONCOMITANT THERAPIES

## Disallowed Concomitant Drug Therapies

Any antimalarial, or antibiotic with antimalarial activity (erythromycin or other macrolides, co-trimoxazole or other sulfonamides, any tetracycline including doxycycline, and quinolones, clindamycin). Intake of these drugs leads to withdrawal of the patient from the further participation in the study.

## Allowed Concomitant Drug Therapies

During the trial patients can be prescribed drugs e.g. paracetamol, and antibiotics with no known antimalarial activity (penicillins, cephalosporins). The dose, route, time and duration of any concomitant medical treatment will recorded in the CRF.

## Special Conditions

Parents or guardians will be discouraged from obtaining drugs from any other source such as private pharmacies, markets or clinics. Parents/guardians will be encouraged to bring their children to the study clinic if their child is unwell or if they are worried about their child's health.

## Rescue Treatments

Patients with clinical treatment failure will be withdrawn from the study, treated, and followed up as per local practice. They will not have study investigations performed thereafter. Patients requiring rescue therapy (ETF, LCF) will be treated by members of the study team with quinine 10 mg/kg orally three times a day for 7 days or any other appropriate oral treatment unrelated to the study drugs. Any patient, who is diagnosed with severe malaria or danger signs during follow-up, will be referred for treatment with parenteral quinine and supportive measures at the local facility or hospital.

Patients with asymptomatic recurrent parasitaemia will also receive rescue therapy.

# PATIENT WITHDRAWAL CRITERIA

During the study, the following conditions are reasons **for stopping the study treatment and/or for excluding the patients from further efficacy assessments:**

1. Any treatment failure (including early failures);

2. Withdrawal of informed consent;

3. Use of antimalarial drugs (or antibiotics with antimalarial activity) outside of the study protocol;

4. Persistent vomiting of study drugs **on Day 0**  (at least twice the same dose);

5. Failure to complete the study treatment

6. Investigator's request for safety reasons (for example, onset of serious adverse events, necessitating study interruption);

6. Lost to follow-up (unable to locate the patient) before Day 28.

Patients with one or more of the conditions listed above will have to be followed-up for the safety assessment until Day 42

These reasons for premature withdrawing will be referred to Day 28 (primary evaluation time).

The reasons for withdrawing between Day 29 and Day 42 will also be recorded in the study CRF.

# PROTOCOL VIOLATIONS

A protocol violation occurs when an event happens that does not allow for accurate interpretation of response to treatment.

Patients meeting one of the following criteria will be considered protocol violations but will not be withdrawn from follow-up:

- **violation of any eligibility criteria;**
- PCR unclassifiable results;
- failure to attend a follow-up visit on Days 0-2 and patient cannot be located within 6 hours of scheduled time;
- failure to attend two consecutive follow-up visits from Day 3 to Day 28 (visits from Day 3 to 28 must take place within 24 hours of the scheduled time).

**The violations listed above are not valid reasons for withdrawing the patients from follow-up (both efficacy and safety assessments). On the contrary, the Investigator must do his/her best to respect the allowed time windows at every visit, even if a violation occurred at a previous visit (i.e. a previous visit did not take place or occurred outside the allowed time window).**

**The protocol violations listed above will not be taken into account for establishing patient inclusion in the Intention-To-Treat population while they will be a reason for excluding the patients from the Evaluable population**.

# SAFETY VARIABLES

Safety and tolerability of the treatments will be evaluated by recording Adverse Events (AEs) and grading, laboratory, ECG and vital signs evaluations.

## Adverse Events

At each visit, the Investigator will ascertain the occurrence of any adverse events since the last visit. Any event must be recorded on the CRF.

### Definition of an adverse event

An AE is any untoward medical occurrence in a patient or clinical investigation subject administered a pharmaceutical product and which does not necessarily have a causal relationship with this treatment.

An AE can therefore be any unfavourable and unintended sign (that could include a clinically significant abnormal laboratory finding), symptom or disease temporally associated with the use of a medicinal product, whether or not considered related to the medicinal product.

### Severity, relationship of event to study drug, and outcome

The severity of a clinical adverse event is to be scored according to the following scale :

1 Mild Awareness of sign or symptom, but easily tolerated

2 Moderate Discomfort enough to cause interference with usual activity

3 Severe Incapacitating with inability to work or perform usual activity

The relationship of an adverse event to study drug is to be assessed according to the following definitions :

1 Definitely unrelated

Should be reserved for those events which occur prior to test drug administration (e.g., washout or single-blind placebo) or for those events which cannot be even remotely related to study participation (e.g. injury caused by a third party).

2 Unlikely

There is no reasonable temporal association between the study drug and the suspected event and the event could have been produced by the subject's clinical state or other modes of therapy administered to the subject.

3 Possible

The suspected adverse event may or may not follow a reasonable temporal sequence from study drug administration but seems to be the type of reaction that cannot be dismissed as unlikely. The event could have been produced or mimicked by the subject's clinical state or by other modes of therapy concomitantly administered to the subject.

4 Probable

The suspected adverse event follows a reasonable temporal sequence from study drug administration, abates upon discontinuation of the drug, and cannot be reasonably explained by the known characteristics of the subject's clinical state.

5 Definitely related

Should be reserved for those events which have no uncertainty in their relationship to test drug administration: this means that a rechallenge was positive.

The outcome of each AE must be assessed according to the following classification:

| - completely recovered : | The patient has fully recovered with no observable residual effects |
| --- | --- |
| - not yet completely recovered : | Improvement in the patient’s condition has occurred, but the patient still has some residual effects |
| - deterioration : | The patient’s overall condition has worsened |
| - permanent damage : | The AE has resulted in a permanent impairment |
| - death : | The patient died due to the AE |
| - ongoing : | The AE has not resolved and remains the same as at onset |
| - unknown : | The outcome of the AE is not known because the patient did not return for follow-up (lost to follow-up) |

### Defintion of a serious adverse event

A serious adverse event (experience) (SAE) or reaction is any untoward medical occurrence that at any dose :

- results in death;
- is life-threatening;
- requires hospitalization (other than for drug administration) or prolongation of existing hospitalization;
- results in persistent or significant disability/incapacity; or
- is a congenital anomaly/birth defect.

And also

- other important medical events (jeopardise the subject or require intervention to prevent one of the other outcomes listed in the definition above).

**All serious adverse events, whether or not deemed drug-related, or expected, must be reported immediately or within 24 hours (one working day), using the Serious Adverse Event Notification Form , by telefax to :**

**Dr Souad Amel Kechairi**

**MDS Pharma Services**

**Safety Group**

**Fax: +33 1 46 90 26 27**

**(Phone: +33 1 46 90 26 65)**

[souad.amel.kechairi@mdsps.com](mailto:souad.amel.kechairi@mdsps.com)

In addition, serious adverse events may be scanned an de-mailed to:

[Souadamel.kechairi@mdsinc.com](mailto:Souadamel.kechairi@mdsinc.com) AND [katie.bucktrout@mdsinc.com](mailto:katie.bucktrout@mdsinc.com) (both parties must be included on the e-mail and the same time lines apply).

Fax should state “Urgent Serious Adverse Event” on cover page.

MDS Pharma Services will be in charge of SAE notifications to relevant Authorities, according to local regulations.

MDS Pharma Services must forward the report to Sigma-Tau to the attention of **Dr Marco Corsi**, **(fax number +39 06 91393757)** within one working day.

A written first follow-up report from the Investigator must be provided to MDS Pharma Services within 5 working days and is to include a full description of the event and sequelae.

All other AEs not fulfilling the criteria of immediate reporting must be recorded on the Case Report Form. This AE information will be collected on a regular basis during the clinical trial by the Clinical Research Associate of MDS Pharma Services.

### Reporting of adverse events

For all adverse events identified, an adverse event report form will be completed.

For each possible adverse event identified and considered as **serious**, a serious adverse event notification form will be completed.

The following information will be recorded for all adverse events:

1. Description of event
2. Date of event onset
3. Date event reported
4. Severity of the event
5. Relationship of the event to study medication
6. Is the event serious ?
7. Initials of the person reporting the event
8. Was the event episodic or intermittent in nature ?
9. Outcome of adverse event
10. Action taken
11. Date event resolved.

A severity grading scale, based on toxicity grading scales developed by the WHO and the National Institutes of Health, Division of Microbiology and Infectious Diseases, will be used to grade severity of all symptoms, physical exam findings, and haemoglobin results (see Appendix I). Any new event, or an event present at baseline that is increasing in severity, will be considered as an adverse event.

## Laboratory Evaluations

Blood samples will be properly labelled with patients' initials, randomisation number, protocol number, the study day and the date the sample is taken. Haematology and Clinical chemistry assessments will be performed locally at sites. Blood samples for clinical chemistry tests will be sent to MDS Pharma Services central laboratory.

All laboratory results will be reported in Standard International Units or in conventional units.

For laboratory analysis, a total volume of blood of about 2 ml x 4 times will be drawn from each child throughout the study. **Sites may draw blood via venopuncture or finger prick.**

*Haematology and Blood Chemistry Tests*

Haemoglobin, , red blood cells count, total white blood cell count, differential count, platelet count, total bilirubin, ALAT, creatinine.

**Sample management in CISM.**

1. **All samples taken from the study participants will be drawn from capillary finger pricking.**
2. **At each participant visit, one drop of blood will be taken for blood slides and in most of the visits for filter paper too.**
3. **For laboratory analysis, a total volume of blood of about 2mL x 4 times will be drawn from each child throughout the study.**
4. **Should any participant become ill during the study, they will be seen at Manhiça’s Hospital outpatients department, where they shall be managed according to the usual procedures. In that case, and only whenever a positive parasitaemia has been detected, an extra blood sample will be taken for filter paper and haematology determination.**
5. **Haematology and biochemistry assessments, as well as blood slide reading for malaria diagnosis will be performed on site.**
6. **Filter paper for *P.falciparum*’s PCR detection and genotyping will be sent to Antwerp (Belgium) where it will be analysed and stored.**
7. **Blood slides will be kept and stored on site until a certain amount is sent for quality control survey to the reference laboratory:**

**MDS Pharma Services**

**Central Lab GmbH**

**Grossmoorbogen 25**

**21079 Hamburg**

**The slides not sent for quality control procedures will be destroyed.**

1. **The samples taken for haematology and biochemistry determinations will not be stored, and therefore subsequently destroyed, once the tests have been performed.**

***Abnormal Laboratory Test Results***

The Investigator will mark in the CRF the laboratory values out of normal ranges and will indicate those of clinical importance. These will be considered as AEs, and the proper AE reporting procedure should be followed by the Investigator.

## Vital Signs

For a comprehensive safety and tolerability evaluation of the study drug, vital signs (systolic and diastolic blood pressure, heart rate) will be recorded at each visit of the study.

The Investigator should mark in the CRF the heart rate and arterial pressure values out of normal ranges and indicate those of clinical importance. These will be considered as AEs, and the proper AE reporting procedure will be followed by the Investigator.

# CASE REPORT FORM (CRF)

Presentation of the CRF

The CRF to be used for the study consists of pages headed with the study code and other relevant information. It is composed of an introductive part for the selection and inclusion of patients in the study and special forms for the different evaluation times; at the end of the CRF are the forms for registration of possible adverse events and for any suspension of the study. Each page includes a header containing information for the identification on the study subject (Study number and Initials; to be completed by the Investigator) and the study code.

How to use the CRF

It is recommended that the CRF be filled out using a ballpoint pen with black ink.

All requested information must be entered on the CRFs. If an item is not available or is not applicable, this fact should be indicated; there should be no blank spaces. A correction should be made by striking through the incorrect entry with a single line and by entering the correct information adjacent to it. The correction must be initialled, dated and explained if necessary by the Investigator or by a qualified individual specifically designated by the Investigator. Each completed Case Report Form must be reviewed, signed and dated by the Investigator.

# STATISTICS

The statistical analysis will be performed by MDS Pharma Services and detailed in the statistical analysis plan (SAP) that will be prepared within the first month of study initiation.

## Population Analysed

The following populations will be considered for the statistical analysis of efficacy.

**Intention-to-Treat (ITT) Population**. Defined as all randomized patients who will take at least one dose of the study treatment. All patients who withdrew from the study for one of the reasons listed in section 5 as well as the patients for whom the PCR is not interpretable or missing will be evaluated as failures. For the robustness analyses, these patients will be evaluated in different ways in this population (see section 9.2).

**Modified Intention-to-Treat (modified ITT) Population**. Defined as the ITT population with the exclusion of the lost-to-follow-up patients before Day 28 for unknown reasons (non informative drop-outs). More specifically, patients withdrawing from the study for reason 7 (see section 5) will be excluded, while all other withdrawals will be counted as failures (these reasons might be potentially considered correlated with lack of efficacy, i.e. are referred to as informative drop-outs). The patients for whom the PCR is not interpretable or missing will be assigned the result “recrudescence” or “re-infection” according to the ratio between these outcomes which was observed among the evaluable PCRs within each treatment group. This rule about PCR will be applied for the primary analysis. For all analyses for which the knowledge of the outcome is needed for each patient individually (for example, the Breslow Day test or the logistic regression or the analyses where a baseline covariate is needed), different scenarios will be considered, i.e. the most two extreme ones of considering all patients with not interpretable or missing PCRs as failures or as successes and the intermediate one of excluding them all.

**Per-Protocol (PP) Population**: defined as all randomized patients who will be eligible according to the study protocol, will receive at least 80% of the study medication, will have the Day 28 assessment, will not take other anti-malarial drugs and, in case of the presence of asexual parasite stages on thick or thin blood smears, will have an evaluable PCR. The protocol violations listed below will also be considered reasons for excluding the patients from this population:

• failure to attend a follow-up visit on Days 0-2 and patient cannot be located within 6 hours of scheduled time;

• failure to attend two consecutive follow-up visits from Day 3 to Day 28 (visits from Day 3 to 28 must take place within 24 hours of the scheduled time and from Days 14 to 28 within 48 hours of the scheduled time).

## Efficacy

9.2.1 Analysis for showing non-inferiority of Artekin vs Coartem

The primary analysis will be based on a 97.5% (one-sided) Confidence Interval (CI) computed on the difference between the 28-day PCR corrected cure rates of the test and the reference treatments, respectively.

In order to claim that Artekin is non-inferior to Coartem the lower limit of the CI must be > -0.05 in both the modified ITT and the Per Protocol populations. The analysis on the more pure ITT population (see section 9.1) will be used for investigating robustness of results (see below).

The method for computing the CI will be specified in the SAP.

For computing the PCR-corrected cure rate, the patients who are diagnosed to be re-infected will be counted as successes (they have to be terminated on that day of follow-up) while the patients for whom the PCR is not interpretable or the sample is unavailable will be treated differently depending on the population considered (see section 9.1).

9.2.2 Historical comparisons

In order to compare the study results with the historical ones, the criteria described in the WHO document (“Assessment and Monitoring of Antimalarial Drug Efficacy for the Treatment of Uncomplicated Falciparum Malaria – 2003”. See chapter 7, section 7.3) will be used. Therefore, the failure rates will be estimated through survival analysis (Kaplan-Meier method) in each treatment group individually considered. This analysis will be carried-out in the per-protocol population and the results obtained in each of the two treatment groups will be judged against the threshold of 90% (there is consensus among the malaria experts in recognising that the threshold of 90% should be considered as a “rule of thumb” for judging efficacy)..

Robust analysis will be performed on the more pure ITT population by applying the following methods:

1. All patients who withdrew from the study for one of the reasons listed above (from 1 to **7**) will be evaluated as failures.

2. Patients who withdrew from the study for reasons that cannot be considered uncorrelated with the study treatments (i.e. reasons from 1 to **6**) will be counted as failures (i.e. assigned with a 0 value), while the patients prematurely interrupting the study for reason **7** will be assigned with a value ranging from 0.5 to 0.9 (imputation of a postulated probability of success for the patients for whom the treatment outcome could not be evaluated). Finally, all cured patients will be assigned with a 1 and this transformed endpoint will be summarized in terms of means and corresponding standard errors by treatment group.

3. The analysis of the 28-day PCR corrected cure rates will be performed by means of the survival analysis techniques, where the non informative lost-to-follow-up patients (i.e. patients withdrawing for unknown reasons) will be censored at the time of exiting the study.

The impact of these strategies on the conclusions will be carefully evaluated and discussed

The Breslow Day test or logistic regression will be used to evaluate homogeneity across centers. In this respect, each center would need to have at least two failures for each treatment in order for large sample chi-square approximations to be applicable. If this type of condition does not apply, exact logistic regression will be used to address homogeneity across centers. In addition, if the sample sizes are sufficiently large within centers so that each treatment has at least five failures within each center, a confidence interval adjusted for centers as strata will be computed (see Koch and others, Statistics in Medicine, 1998).

## Efficacy: secondary analysis

All the secondary statistical analysis will be detailed in the statistical analysis plan (SAP).

## Safety and Tolerability

All randomised patients who have taken at least one dose of the study medications will be considered for the following analysis.

All Adverse Events (AEs) will be tabulated according to degree of seriousness, time of treatment at AE onset, severity, relation to study drug, action taken, if any, and final outcome.

Vitals signs and ECG will be analysed descriptively.

ECG results will be classified as normal and abnormal. The evaluation of ECG and QT interval will be done according to the EMEA directive “point to consider: the assessment of the potential for QT interval prolongation by non-cardiovascular medicinal products”. EMEA (CPMP), London 17/12/1997 (CPMP/986/96).

# MONITORING AND QUALITY ASSURANCE

The task of the Monitor is to verify the best conduct of the study through frequent contacts by phone and in person with the Principal Investigator and site staff, in accordance with the Standard Operating Procedures and Good Clinical Practice, with the purposes of facilitating the work and attaining the objectives of the study. These visits will enable the Monitor to maintain current, personal knowledge of the study through review of the records, comparison with source documents, observation and discussion of the conduct of the study with the Investigator.

The Investigator by signing this protocol declares that he/she will permit trial-related monitoring, audits, Independent Ethic Committee review, and regulatory inspections, providing direct access to source data/documents.

The Investigator agrees to conduct the present study in full agreement with the principles of the “Declaration of Helsinki” and subsequent relevant amendments (see Appendix III).

# DATA MANAGEMENT

All data management procedures will be detailed in separate, specifically identified files that collectively will be referenced as the Data Management Plan (DMP).

Appropriate CRFs will be prepared for collection of data requested by the protocol. All response variables will be entered into an electronic database maintained by MDS Pharma services. This database will be compiled and reviewed for accuracy in accordance with MDS Pharma services internal Standard Operating Procedures concerning data management and data quality control. All data will be tabulated to generate data listings for the final report.

These data will be then exported for the creation of Statistical Analysis System (SAS®) datasets that comprise the SAS® database. Edit and consistency checks will also be performed as outlined in the DMP. Once all questions have been resolved, the database will be locked. The locked SAS® database will be used to generate the subject listings, tabulations, and analyses.

# INVESTIGATOR RESPONSIBILITY

Except where the Principal Investigator's signature is specifically required, it is understood that the term "Investigator" as used in this protocol and on the CRFs refers to the Principal Investigator or a member of the staff that the Investigator designates to perform a certain duty under this protocol. The Investigator is ultimately responsible for the conduct of all aspects of the study.

For all other relevant Investigator responsibilities see “CPMP/ICH/135/95 Topic E6 - Guideline for Good Clinical Practice”, Chapter 4.

# TRIAL REPORTS

## Statistical Report

The Statistical Report will be prepared by the Biostatistics and Data Management Department of MDS Pharma Services after having received all the validated CRFs.

## Final Clinical Report

The Final Clinical Report will be written by MDS Pharma Services. It is structured as an Integrated Clinical Report containing clinical comments based on the data generated by the Statistical Report.

# ADMINISTRATIVE PROCEDURES

## Regulatory Authorities and Ethical Review Committee

This study will be reported to the respective National Health Authority.

The clinical protocol will be submitted for approval to the relevant Independent Ethic Committee (IEC) or Institutional Review Board (IRB) before patients can be enrolled.

Copy of the IEC/IRB approval will be transmitted from the Investigator to the Sponsor before starting the study.

## Informed Consent

All interviews will be conducted in the native language of the patients by the study personnel. Consent forms will be provided to the parents or guardians for their review (see Appendix IV). The parents or guardians will be asked to sign consent to participate in a research study. The informed consent will describe the purpose of the study, the procedures to be followed, and the risks and benefits of participation. If a parent or guardian is unable to read or write, a signature from a witness to the informed consent discussion will be obtained. Parents or guardians will be informed that participation in the study is completely voluntary and that they may withdraw their child from the study at any time without any negative consequences.

## Confidentiality and Publication of Results

All study documents are provided by the Sponsor in confidence to the investigators and his/her appointed staff. None of this material may be disclosed to any part not directly involved in the study without written permission from Sigma-Tau i.f.r. S.p.A., Pomezia (Rome) – Italy.

Presentation and publication of the study results will be carried out by Investigators jointly with the Sponsor (i.e.: Sigma-Tau), that will be informed at least 3 months before disclosure of the data, in order to discuss the content of the presentation or manuscript.

## Protocol Amendments

Once the final clinical protocol has been issued and signed by the Investigator and the authorised signatories, it must not be informally altered. Clinical protocol amendments are alterations to a legal document (the clinical protocol) and have the same legal status and must pass through the appropriate steps before being implemented. In general, any change must be approved by the IEC prior to be effective. Administrative changes need only notification to the IEC without approval.

Any subsequent amendments must be made on separate sheet and must pass through the approval process. It must be clear to the Investigator that he can not change the clinical protocol without prior discussion with Sponsor, which should give its approval.

# Study Committees

## Steering Committee

The Steering Committee (SC) comprises at least one investigator from each participating region and will assess the progress of the trial. The members of the SC will address policy and operational issues related to the protocol. The SC has responsibility for protecting the scientific conduct and integrity of the trial. Its functions include:

- Review of the protocol before ethic committee approval,
- Formulation of recommendation for any change in the design and operations of the trial during the course of the trial, when needed,
- Exclusion of patients from the per protocol analysis.

The members of the Steering Committee will be specified in the Terms of Agreement document that will be prepared no later than the first face-to-face meeting of this Committee

## Data Monitoring Board

The Data Monitoring Board (DMB) will be composed of one statistician and two or three clinicians, all of them independent from the Sponsor and its Designee. At least one of the clinicians will have experience in treating patients with the disease under study and at least one will be a malaria expert.

The main roles of the DMB will be:

• performing the interim analysis for sample size re-assessment;

• monitoring all safety data recorded while the study is ongoing;

• revising in blind a significant percentage (to be defined in the SAP) of the primary efficacy data.

The members of the DMB will be identified prior to enrolling the first patient.

**15.3 Clinical Development Committee**

The Clinical Development Committee will be responsible for:

- evaluating the recommendations of both the Data Monitoring Committee and the study Steering Committee and, if deemed appropriate, transforming them in operational decisions;
- protecting the scientific conduct, the ethical integrity, and the regulatory acceptability of the Artekin project;
- harmonizing all the studies of the Artekin project;
- addressing policies and operational issues that can impact the Artekin project.

The members appointed to this Committee will be specified in the Terms of Agreement document that will be prepared no later than the first face-to-face meeting of this Committee

# REFERENCES

1. **Olliaro P. L. and Taylor W. R. J.**

Review. Antimalarial compounds: from bench to bedside.

*J. Experim. Biol.****206****, 3753-3759, 2003*

2. **World Health Organization**

Antimalarial Drug Combination Therapy. Report of a WHO Technical Consultation. WHO/CDS/RBM/ 2001.35.Geneva, Switzerland: World Health Organization.

3. **White N. J.**

Antimalarial drug resistance: the pace quickens.

*J. Antimicrob. Chemother.* ***30****, 571-585, 1992*

4. **White, N. J.**

Delaying antimalarial drug resistance with combination chemotherapy.

*Parasitologia* ***41****, 301-308, 1999*

5. **White, N. J., Nosten, F., Looareesuwan, S., Watkins, W. M., Marsh, K., Snow, R. W., Kokwaro, G., Ouma, J., Hien, T. T., Molyneux, M. E. et al.**

*Averting a malaria disaster. Lancet* ***353****, 1965-1967, 1999*

6. **White, N. J.**

Assessment of the pharmacodynamic properties of antimalarial drugs in vivo.

*Antimicrob. Agents Chemother.* ***41****, 1413-1422, 1997*

7. **Kumar, N. and Zheng, H.**

Stage-specific gametocytocidal effect in vitro of the antimalaria drug qinghaosu on Plasmodium *falciparum*.

*Parasitol. Res. 7****6****, 214-218, 1990*

8. **Targett, G., Drakeley, C., Jawara, M., von Seidlein, L., Coleman, R., Deen, J., Pinder, M., Doherty, T., Sutherland, C., Walraven, G. et al.**

Artesunate reduces but does not prevent posttreatment transmission of Plasmodium *falciparum* to *Anopheles gambiae*.

*J. Infect. Dis.* ***183****, 1254-1259, 2001*

9. **Price, R. N., Nosten, F., Luxemburger, C., ter Kuile, F. O., Paiphun, L., Chongsuphajaisiddhi, T. and White, N. J.**

Effects of artemisinin derivatives on malaria transmissibility.

*Lancet* ***347****, 1654-1658, 1996*

10. **Nosten, F., van Vugt, M., Price, R., Luxemburger, C., Thway, K. L., Brockman, A., McGready, R., ter Kuile, F., Looareesuwan, S. and White, N. J.**

Effects of artesunate-mefloquine combination on incidence of Plasmodium falciparum malaria and Mefloquine resistance in western Thailand: a prospective study.

*Lancet* ***356****, 297-302, 2000*

11. **White, N. J. and Olliaro, P.**

Artemisinin and derivatives in the treatment of uncomplicated malaria.

*Med. Trop.* ***58****(Suppl), 54-56, 1998*

12. **McGready, R., Cho, T., Keo, N. K., Thwai, K. L., Villegas, L., Looareesuwan, S., White, N. J. and Nosten, F.**

Artemisinin antimalarials in pregnancy: a prospective treatment study of 539 episodes of multidrug-resistant Plasmodium *falciparum*.

*Clin. Infect. Dis.* ***33****, 2009-2016, 2001*

13. **Trouiller, P., Olliaro, P., Torreele, E., Orbinski, J., Laing, R. and Ford, N.**

Drug development for neglected diseases: a deficient market and a public-health policy failure.

*Lancet* ***359****, 2188-2194, 2002*

14. **Sulo, J., Chimpeni, P., Hatcher, J., Kublin, J. G., Plowe, C. V., Molyneux, M. E., Marsh, K., Taylor, T. E., Watkins, W. M. and Winstanley, P. A.**

Chlorproguanil-dapsone versus sulfadoxine-pyrimethamine for sequential episodes of uncomplicated falciparum malaria in Kenya and Malawi: a randomised clinical trial.

*Lancet* ***360****, 1136-1143, 2002*

15. **Mutabingwa, T., Nzila, A., Mberu, E., Nduati, E., Winstanley, P., Hills, E. and Watkins, W.**

Chlorproguanil-dapsone for treatment of drug resistant falciparum malaria in Tanzania.

*Lancet* ***358****, 1218-1223, 2001*

16. **Omari, A. A., Preston, C. and Garner, P.**

Artemether-lumefantrine for treating uncomplicated falciparum malaria.

*Cochrane Database Syst. Rev.* ***2****, CD003125, 2003*

17. **Vugt, M. V., Wilairatana, P., Gemperli, B., Gathmann, I., Phaipun, L., Brockman, A., Luxemburger, C., White, N. J., Nosten, F. and Looareesuwan, S.**

Efficacy of six doses of artemether-lumefantrine (benflumetol) in multidrug-resistant Plasmodium *falciparum* malaria.

*Am. J. Trop. Med. Hyg.* ***60****, 936-942, 1999*

18. **van Vugt, M., Looareesuwan, S., Wilairatana, P., McGready, R., Villegas, L., Gathmann, I., Mull, R., Brockman, A., White, N. J. and Nosten, F.**

Artemether-lumefantrine for the treatment of multidrug-resistant falciparum malaria.

*Trans. R. Soc. Trop. Med. Hyg.* ***94****, 545-548, 2000*

19. **Ringwald, P., Bickii, J. and Basco, L.**

Randomised trial of pyronaridine versus chloroquine for acute uncomplicated falciparum malaria in Africa.

*Lancet* ***347****, 24-28, 1996*

20. **Looareesuwan, S., Kyle, D. E., Viravan, C., Vanijanonta, S., Wilairatana, P. and Wernsdorfer, W. H.**

Clinical study of pyronaridine for the treatment of acute uncomplicated falciparum malaria in Thailand. *Trans. R.*

*Soc. Trop. Med. Hyg.* ***54****, 205-209, 1996*

21. **S. Looareesuwan, V. Navaratnam and P. L. Olliaro**

Unpublished, personal communication.

22. **Lell, B., Ruangweerayut, R., Wiesner, J., Missinou, M. A., Schindler, A., Baranek, T., Hintz, M., Hutchinson, D., Jomaa, H. and Kremsner, P.G.**

Fosmidomycin, a novel chemotherapeutic agent for malaria.

*Antimicrob. Agents Chemother.* ***47****, 735-738, 2003*

23. **Missinou, M. A., Borrmann, S., Schindler, A., Issifou, S., Adegnika, A. A., Matsiegui, P. B., Binder, R., Lell, B., Wiesner, J., Baranek, T. et al.**

Fosmidomycin for malaria.

*Lancet* ***360****, 1941-1942, 2002*

24. **Denis, M. B., Davis, T. M., Hewitt, S., Incardona, S., Nimol, K., Fandeur, T., Poravuth, Y., Lim, C. and Socheat, D.**

Efficacy and safety of dihydroartemisinin-piperaquine (Artekin) in Cambodian children and adults with uncomplicated falciparum malaria.

*Clin. Infect. Dis.* ***35****, 1469-1476, 2002*

25. **Wilairatana, P., Krudsood, S., Chalermut, K., Pengroska, C., Srivilairit, S., Silachamroon, U., Treeprasertsuk, S. and Looareesuwan, S.**

An open randomized clinical trial of Artecom vs artesunate-mefloquine in the treatment of acute uncomplicated falciparum malaria in Thailand.

*Southeast Asian J Trop. Med. Public Health* ***33(3),*** *519-524, 2002*

26. **Hien, T.T., Dolecek, C., Mai, P.P., Dung, N.T., Truong, N.T., Thai, L.H., An, D.T.A., Thanh, T.T., Stepniewska, K., White, N., and Farrar, J.**

Dihydroartemisinin-piperaquine against multidrug-resistant Plasmodium *falciparum* malaria in Vietnam: randomized clinical trial.

*Lancet* ***363****, 18-22, 2004*

27. **Karunajeewa, H., Lim, C., Hung, T.Y., Ilett, K.F., Denis, M.B., Socheat, D. and Davis, T.M.E.**

Safety evaluation of fixed combination piperaquine plus dihydroartemisinin (Artekin) in Cambodian children and adults with malaria.

*Br. J. Clin. Pharmacol. 57, 93-99, 2003*

28. **Hung, T.Y., Davis, T.M.E., Ilett, K.F., Karunajeewa, H., Hewitt, S., Denis, M.B., Lim, C. and Socheat, D.**

Population pharmacokinetics of piperaquine in adults and children with uncomplicated falciparum or vivax malaria.

*Br. J. Clin. Pharmacol. 57:3, 253-262, 2003*

29. **Ashley, E.A., Krudsood, S., Phaiphun, L., Srivilairit, S., McGready, R., Leowattana, W., Hutagalung, R., Wilairatana, P., Brockman, A., Looareesuwan, S., Nosten, F., White, N.J.**

Dose optimization randomized controlled studies of dihydroartemisinin-piperaquine for the treatment of uncomplicated multi-drug resistant falciparum malaria in Thailand.

*Personal communication, JID 2004: 190 (15 November), 1773-82*

30. **Myint,H., Ashley, E. and White, N.**

Reported adverse effects of dihydroartemisinin-piperaquine (Artekin).

*Personal communication, 2004*

31. **Danis, M., Bricaire, F.**

The new drug combinations: their place in the treatment of uncomplicated Plasmodium falciparum malaria.

*Fundam. Clin. Pharmacol.* ***17****, 155-160, 2003*

32. **Premji Z, Makanga M, Falade C et al.**

Efficacy, safety and pharmacokinetics of a co-artemether (Artemether 20 mg, Lumefantrine 120 mg), 6-dose regimen, in African infants and children with acute, uncomplicated plasmodium falciparum malaria".

*Symposium " Overcoming Challenges to Change" (7 February 2004, Cape Town, South Africa).*

33. **von Seidlein L, Jeffer S, Pinder M et al.**

Treatment of African children with uncomplicated falciparum malaria with a new antimalarial drug CGP 56697.

*The Journal of Infectious Diseases* ***176****: 1113-1116, 1997*

34. **Gould AL.**

Interim analysis for monitoring clinical trials that do not materially affect the type I error rate.

*Statistics in Medicine* ***11****: 55-66, 1992*

35. **Herson J, Wittes J.**

The use of interim analysis for sample size adjustment*.*

*Drug Information Journal* ***27****: 753-760, 1993*

# APPENDICES

**APPENDIX I**

Guidelines for Grading Patient Symptoms, signs and laboratory findings.

Table A. Guidelines for Grading Patient Symptoms.

|  | **Grade 1**  **MILD** | **Grade 2**  **MODERATE** | **Grade 3**  **SEVERE** | **Grade 4**  **LIFE THREATENING** |
| --- | --- | --- | --- | --- |
| **Subjective fever in the past 24 h** | N/A | Present (Yes) | N/A | N/A |
| ***Weakness*** | Mild decrease in activity; For children – weak, but still playing | Moderate decrease in activity; For children – weak, and playing limited | Not participating in usual activities; For children – not playing | Prostration |
| **Muscle and/or joint aches*** | Mild and/or localized complaints | Diffuse complaints | Objective weakness; function limited | N/A |
| **Headache*** | Mild, no treatment required | Transient, moderate; treatment required | Severe, constant; requires narcotic therapy | Intractable; requires repeated narcotic therapy |
| **Anorexia** | Decreased appetite, but still taking solid food | Decreased appetite, avoiding solid food but taking liquids | Appetite very decreased; Refusing to breast feed, no solids or liquids taken (< 2 years < 12 hr; > 2 years < 24 hr) | Appetite very decreased; Refusing to breast feed, no solids or liquids taken (< 2 years > 12 hr; > 2 years > 24 hr) |
| **Nausea*** | Mild, transient feeling of impending vomiting; maintains reasonable intake | Moderate and/or constant feeling of impending vomiting; intake decreased | Severe, constant feeling of impending emesis; intake decreased significantly | N/A |
| **Vomiting** | 1 episode per day | 2-3 episodes per day | Orthostatic hypotension or IV fluids required | Hypotensive shock or physiological consequences requiring IV fluid therapy |
| **Abdominal pain*** | Mild (1-3 on a scale of 1 to 10) | Moderate (4-6 on a scale of 1 to 10) | Moderate to severe (> 7 on a scale of 1 to 10) | Severe – hospitalization for treatment |
| **Diarrhea** | Transient 3-4 loose stools/day | 5-7 loose stools/day | Orthostatic hypotension or > 7 loose stools/day or IV fluids required | Hypotensive shock or physiological consequences requiring IV fluid therapy |
| **Cough** | Transient / intermittent | Persistent / constant | Uncontrolled | Cyanosis, stridor, severe shortness of breath |
| **Pruritis** | Transient pruritis | Pruritis that disturbs sleep | Severe, constant pruritis, sleep disturbed | N/A |
| **Tinnitus*** | Mild, transient ringing or roaring sound | Moderate, persistent ringing or roaring sound | Severe ringing or roaring sound with associated hearing loss | N/A |
| **Behavioural changes** | Mild difficulty concentrating; mild confusion or agitation; activities of daily living unaffected; no treatment | Moderate confusion or agitation; some limitation of activities of daily living; minimal treatment | Severe confusion or agitation; Needs assistance for activities of daily living; therapy required | Toxic psychosis; hospitalization for treatment |
| **“Flu”**  **(viral URI)** | Mild nasal congestion, mild rhinorrhea | Moderate nasal congestion, moderate rhinorrhea | N/A | N/A |
| **Allergic reaction** | N/A | N/A | Urticaria | Severe urticaria  anaphylaxis, angioedema |
| **Convulsion** | N/A | N/A | Localized or generalized seizure | Status epilepticus |
| *** Assess only in children > 3 years of age. Answer N/A for younger children and those unable to answer.** | | | | |

Reference – Based on WHO Toxicity Grading Scale for Determining the Severity of Adverse Events

**Table B. Guidelines for Physical Examination**

| **Dehydration** | Assess skin touch and turgor, mucous membranes, eyes, crying, fontanelle, pulse, urine output |
| --- | --- |
| **Jaundice** | Assess for yellowing of the sclera. Also evaluate the palpebral conjunctiva, lips, and skin. |
| **Chest** | Observe the rate, rhythm, depth, and effort of breathing. Check the patient’s colour for cyanosis.  The maximum acceptable respiratory rate by age: < 2 months = 60, 2-12 months = 50, 1-5 years = 40, above 5 years = 30.  Inspect the neck for the position of the trachea, for supraclavicular retractions, and for contraction of the sternomastoid or other accessory muscles during inspiration.  Auscultate the anterior and posterior chest for normal breath sounds and any adventitious sounds (crackles or rales, wheezes, and rhonchi). *Crackles are intermittent, non-musical, fine or coarse sounds that may be due to abnormalities of the lungs (pneumonia, fibrosis, early congestive heart failure) or airways (bronchitis or bronchiectasis). Wheezes are high-pitched and result from narrowed airways. Rhonchi are relatively low-pitched and suggest secretions in large airways.*  If abnormalities are identified, evaluate for transmitted voice sounds. In addition, palpate the chest to assess for tactile fremitus, and percuss the chest to assess for areas of dullness*. Normal, air-filled lungs emit predominantly vesicular breath sounds, transmit voice sounds poorly with “ee” = “ee”, and have no tactile fremitus. Airless lung, as in lobar pneumonia, emits bronchial breath sounds, transmits spoken words clearly with “ee” = “aay” (egophany), and has an increase in tactile fremitus.* |
| **Abdomen** | Inspect and ausculate the abdomen. Listen for bowel sounds in the abdomen before palpating it. Palpate the abdomen in all 4 quadrants lightly and then deeply. Assess the size of the liver and spleen. To assess for peritoneal inflammation, look for localised and rebound tenderness, and voluntary or involuntary rigidity. |
| **Skin** | Inspect the skin for colour, turgor, moisture, and lesions. If lesions are present, note their location and distribution (diffuse or localised), arrangement (linear, clustered, annular, dermatomal), type (macules, papules, vesicles) and colour. |
| **Tablet test** | For children > 9 months of age, ask the patient to pick a tablet (or equivalent object) up off a flat surface using the thumb and index finger of their dominant hand*. This tests for co-ordination of the upper extremity assessing the function of the motor system, cerebellar system, vestibular system (for coordinating eye and body movements) and the sensory system, for position sense. When testing small children, be aware that they will likely attempt to put the object into their mouth.* |

**Table C. Grading Physical Examination Findings**

|  | **Grade 1**  **MILD** | **Grade 2**  **MODERATE** | **Grade 3**  **SEVERE** | **Grade 4**  **LIFE-THREATENING** |
| --- | --- | --- | --- | --- |
| **Temperature* (axillary)** | 37.5-37.9°C | 38.0-39.5°C | > 39.5°C | Sustained fever, equal or greater than 40.0°C for longer than 5 days |
| **Dehydration** | Less than 2 of the following:  Restless, irritable  Sunken eyes  Drinks eagerly, thirsty  Skin pinch goes back slowly | 2 of the following:  Restless, irritable  Sunken eyes  Drinks eagerly, thirsty  Skin pinch goes back slowly | Two of the following:  Lethargic or unconscious  Sunken eyes  Not able to drink or drinking poorly  Skin pinch goes back very poorly | Two of the following + shock:  Lethargic or unconscious  Sunken eyes  Not able to drink or drinking poorly  Skin pinch goes back very poorly |
| **Jaundice** | Slight yellowing of sclera and conjunctiva | Moderate yellowing of sclera and conjunctiva, yellowing of mucous membranes | Severe yellowing of sclera and conjunctiva, yellowing of skin | N/A |
| **Chest** | Mildly increased RR (for age, temperature), transient or localised adventitious sounds | Moderately increased RR, diffuse or persistent adventitious sounds | Rapid RR (< 2 months > 60, 2-12 months > 50, 1-5 years > 40, adults > 30)* nasal flaring, retractions | Cyanosis |
| **Abdomen** | Normal bowel sounds, mild localised tenderness, and/or liver palpable 2-4 cm below the right costal margin (RCM), and/or spleen palpable, and/or umbilical hernia present | Normal or mildly abnormal bowel sounds, moderate or diffuse tenderness; and/or mild to moderately enlarged liver (4-6 cm below the RCM) and/or spleen palpable up to half-way between umbilicus and symphysis pubis | Severely abnormal bowel sounds, severe tenderness to palpation. Evidence of peritoneal irritation and/or significant enlargement of liver (> 6 cm below the RCM) and/or spleen palpable beyond half-way between umbilicus and symphysis pubis | Absent bowel sounds. Involuntary rigidity |
| **Skin†** | Localised rash, erythema, or pruritis | Diffuse, maculopapular rash, dry desquamation | Vesiculation, moist desquamation, or ulceration | Exfoliative dermatitis, mucous membrane involvement or erythema multiforme or suspected Stevens-Johnson or necrosis requiring surgery |

|  | **Grade 1**  **MILD** | **Grade 2**  **MODERATE** | **Grade 3**  **SEVERE** | **Grade 4**  **LIFE-THREATENING** |
| --- | --- | --- | --- | --- |
| **Hearing** | *< 4 years: N/A*  > 4 years: Decreased hearing in one ear | *< 4 years: N/A*  > 4 years: Decreased hearing in both ears or severe impairment in one ear | *< 4 years: Any evidence of hearing impairment*  > 4 years: Severe impairment in both ears | N/A |
| **Tablet test** | Difficulty grasping tablet but able to pick up | Unable to pick up tablet without dropping | Unable to grasp tablet | N/A |
| **Clinical symptoms / sign *(not otherwise specified)*** | No treatment required; monitor condition | Treatment required | Requires treatment and possible hospitalisation | Requires active medical intervention, hospitalisation, or hospice care |

Reference – The Harriet Lane Handbook, 15th edition, 2000

† Reference – WHO Toxicity Grading Scale for Determining the Severity of Adverse Events

**TABLE D. Guidelines for Grading of Laboratory Abnormalities**

|  | **Grade 1**  **MILD** | **Grade 2**  **MODERATE** | **Grade 3**  **SEVERE** | **Grade 4**  **LIFE-THREATENING** |
| --- | --- | --- | --- | --- |
| **Haemoglobin**  ***(****g/dL)* | 9.0 – 9.9 | 7.0 – 8.9 | 5.0 – 6.9 | < 5.0 |

Reference – The Harriet Lane Handbook, 15^th^ edition, 2000†

Reference – WHO Toxicity Grading Scale for Determining the Severity of Adverse Events

**APPENDIX II**

**Table A. Dihydroartemisinin will be given daily. The number of tablets per day is reported in brackets. One tablet of Artekin (pediatric tablets) contains 20/40 mg of DHA and 160/320 mg of PPQ..**

| **Weight in kg** | **mg of DHA to be given daily** | **Dose of DHA as mg/kg/d** |
| --- | --- | --- |
| 4 - 6 | 10 (1/2 tablet with 20 mg) | 1.67 - 2.5 |
| 7 - 12 | 20 (1 tablet with 20 mg) | 1.67 - 2.86 |
| 13 - 23 | 40 (1 tablet with 40 mg) | 1.74 - 3.08 |
| 24 - 35 | 80 (2 tablets with 40 mg) | 2.29 - 3.33 |
| 36 - 75 | 120 (3 tablets with 40 mg) | 1.6 - 3.64 |

*One tablet of Artekin contains 40 mg of DHA and 320 mg of PPQ for adult patients.*

**Table B: Piperaquine will be given daily. The number of tablets per day is reported in brackets. One tablet of Artekin (pediatric tablets) contains 20/40 mg of DHA and 160/320 mg of PPQ.**

| **Weight in kg** | **mg of PPQ to be given daily** | **Dose of PPQ as mg/kg/d** |
| --- | --- | --- |
| 4 - 6 | 80 (1/2 tablet with 160 mg) | 13.36 - 20 |
| 7 - 12 | 160 (1 tablet with 160 mg) | 13.36 – 22.88 |
| 13 - 23 | 320 (1 tablet with 320 mg) | 13.92 – 24.64 |
| 24 - 35 | 640 (2 tablets with 320 mg) | 18.32 – 26.4 |
| 36 - 75 | 960 (3 tablets with 320 mg) | 12.8 – 29.12 |

*One tablet of Artekin contains 40 mg of DHA and 320 mg of PPQ for adult patients.*

**Table C: Coartem® dose based on boy weight will be given daily. Tablets containing 20 mg of Artemether and 120 mg of Lumefantrine.**

| **Weight in kg** | **Number of tablet per dose** |
| --- | --- |
| 5 to < 15 kg | 1 tablet per dose |
| 15 to < 25 kg | 2 tablets per dose |
| 25 to < 35 kg | 3 tablets per dose |

**APPENDIX III**

**WORLD MEDICAL ASSOCIATION DECLARATION OF HELSINKI**

**Ethical Principles for Medical Research Involving Human Subjects**

**Recommendations guiding medical physicians**

**in biomedical research involving human subjects**

Adopted by the 18th WMA General Assembly

Helsinki, Finland, June 1964

and amended by the

29th WMA General Assembly, Tokyo, Japan, October 1975

35th WMA General Assembly, Venice, Italy, October 1983

41st WMA General Assembly, Hong Kong, September 1989

48th WMA General Assembly, Somerset West, Republic of South Africa, October 1996

and the

52nd WMA General Assembly, Edinburgh, Scotland, October 2000

Note of Clarification on Paragraph 29 added by the WMA General Assembly, Washington 2002.

**A. INTRODUCTION**

1. The World Medical Association has developed the Declaration of Helsinki as a statement of ethical principles to provide guidance to physicians and other participants in medical research involving human subjects. Medical research involving human subjects includes research on identifiable human material or identifiable data.

2. It is the duty of the physician to promote and safeguard the health of the people. The physician's knowledge and conscience are dedicated to the fulfillment of this duty.

3. The Declaration of Geneva of the World Medical Association binds the physician with the words, "The health of my subject will be my first consideration," and the International Code of Medical Ethics declares that, "A physician shall act only in the subject's interest when providing medical care which might have the effect of weakening the physical and mental condition of the subject."

4. Medical progress is based on research which ultimately must rest in part on experimentation involving human subjects.

5. In medical research on human subjects, considerations related to the well-being of the human subject should take precedence over the interests of science and society.

6. The primary purpose of medical research involving human subjects is to improve prophylactic, diagnostic and therapeutic procedures and the understanding of the etiology and pathogenesis of disease. Even the best proven prophylactic, diagnostic, and therapeutic methods must continuously be challenged through research for their effectiveness, efficiency, accessibility and quality.

7. In current medical practice and in medical research, most prophylactic, diagnostic and therapeutic procedures involve risks and burdens.

8. Medical research is subject to ethical standards that promote respect for all human beings and protect their health and rights. Some research populations are vulnerable and need special protection. The particular needs of the economically and medically disadvantaged must be recognized. Special attention is also required for those who cannot give or refuse consent for themselves, for those who may be subject to giving consent under duress, for those who will not benefit personally from the research and for those for whom the research is combined with care.

9. Research Investigators should be aware of the ethical, legal and regulatory requirements for research on human subjects in their own countries as well as applicable international requirements. No national ethical, legal or regulatory requirement should be allowed to reduce or eliminate any of the protections for human subjects set forth in this Declaration.

**B. BASIC PRINCIPLES FOR ALL MEDICAL RESEARCH**

10. It is the duty of the physician in medical research to protect the life, health, privacy, and dignity of the human subject.

11. Medical research involving human subjects must conform to generally accepted scientific principles, be based on a thorough knowledge of the scientific literature, other relevant sources of information, and on adequate laboratory and, where appropriate, animal experimentation.

12. Appropriate caution must be exercised in the conduct of research which may affect the environment, and the welfare of animals used for research must be respected.

13. The design and performance of each experimental procedure involving human subjects should be clearly formulated in an experimental protocol. This protocol should be submitted for consideration, comment, guidance, and where appropriate, approval to a specially appointed ethical review committee, which must be independent of the Investigator, the Sponsor or any other kind of undue influence. This independent committee should be in conformity with the laws and regulations of the country in which the research experiment is performed. The committee has the right to monitor ongoing trials. The researcher has the obligation to provide monitoring information to the committee, especially any serious adverse events. The researcher should also submit to the committee, for review, information regarding funding, Sponsors, institutional affiliations, other potential conflicts of interest and incentives for subjects.

14. The research protocol should always contain a statement of the ethical considerations involved and should indicate that there is compliance with the principles enunciated in this Declaration.

15. Medical research involving human subjects should be conducted only by scientifically qualified persons and under the supervision of a clinically competent medical person. The responsibility for the human subject must always rest with a medically qualified person and never rest on the subject of the research, even though the subject has given consent.

16. Every medical research project involving human subjects should be preceded by careful assessment of predictable risks and burdens in comparison with foreseeable benefits to the subject or to others. This does not preclude the participation of healthy volunteers in medical research. The design of all studies should be publicly available.

17. Physicians should abstain from engaging in research projects involving human subjects unless they are confident that the risks involved have been adequately assessed and can be satisfactorily managed. Physicians should cease any investigation if the risks are found to outweigh the potential benefits or if there is conclusive proof of positive and beneficial results.

18. Medical research involving human subjects should only be conducted if the importance of the objective outweighs the inherent risks and burdens to the subject. This is especially important when the human subjects are healthy volunteers.

19. Medical research is only justified if there is a reasonable likelihood that the populations in which the research is carried out stand to benefit from the results of the research.

20. The subjects must be volunteers and informed participants in the research project.

21. The right of research subjects to safeguard their integrity must always be respected. Every precaution should be taken to respect the privacy of the subject, the confidentiality of the subject's information and to minimize the impact of the study on the subject's physical and mental integrity and on the personality of the subject.

22. In any research on human beings, each potential subject must be adequately informed of the aims, methods, sources of funding, any possible conflicts of interest, institutional affiliations of the researcher, the anticipated benefits and potential risks of the study and the discomfort it may entail. The subject should be informed of the right to abstain from participation in the study or to withdraw consent to participate at any time without reprisal. After ensuring that the subject has understood the information, the physician should then obtain the subject's freely-given informed consent, preferably in writing. If the consent cannot be obtained in writing, the non-written consent must be formally documented and witnessed.

23. When obtaining informed consent for the research project the physician should be particularly cautious if the subject is in a dependent relationship with the physician or may consent under duress. In that case the informed consent should be obtained by a well-informed physician who is not engaged in the investigation and who is completely independent of this relationship.

24. For a research subject who is legally incompetent, physically or mentally incapable of giving consent or is a legally incompetent minor, the Investigator must obtain informed consent from the legally authorized representative in accordance with applicable law. These groups should not be included in research unless the research is necessary to promote the health of the population represented and this research cannot instead be performed on legally competent persons.

25. When a subject deemed legally incompetent, such as a minor child, is able to give assent to decisions about participation in research, the Investigator must obtain that assent in addition to the consent of the legally authorized representative.

26. Research on individuals from whom it is not possible to obtain consent, including proxy or advance consent, should be done only if the physical/mental condition that prevents obtaining informed consent is a necessary characteristic of the research population. The specific reasons for involving research subjects with a condition that renders them unable to give informed consent should be stated in the experimental protocol for consideration and approval of the review committee. The protocol should state that consent to remain in the research should be obtained as soon as possible from the individual or a legally authorized surrogate.

27. Both authors and publishers have ethical obligations. In publication of the results of research, the Investigators are obliged to preserve the accuracy of the results. Negative as well as positive results should be published or otherwise publicly available. Sources of funding, institutional affiliations and any possible conflicts of interest should be declared in the publication. Reports of experimentation not in accordance with the principles laid down in this Declaration should not be accepted for publication.

**C. ADDITIONAL PRINCIPLES FOR MEDICAL RESEARCH COMBINED WITH MEDICAL CARE**

28. The physician may combine medical research with medical care, only to the extent that the research is justified by its potential prophylactic, diagnostic or therapeutic value. When medical research is combined with medical care, additional standards apply to protect the subjects who are research subjects.

29. The benefits, risks, burdens and effectiveness of a new method should be tested against those of the best current prophylactic, diagnostic, and therapeutic methods. This does not exclude the use of placebo, or no treatment, in studies where no proven prophylactic, diagnostic or therapeutic method exists.

30. At the conclusion of the study, every subject entered into the study should be assured of access to the best proven prophylactic, diagnostic and therapeutic methods identified by the study.

31. The physician should fully inform the subject which aspects of the care are related to the research. The refusal of a subject to participate in a study must never interfere with the subject-physician relationship.

32. In the treatment of a subject, where proven prophylactic, diagnostic and therapeutic methods do not exist or have been ineffective, the physician, with informed consent from the subject, must be free to use unproven or new prophylactic, diagnostic and therapeutic measures, if in the physician's judgment it offers hope of saving life, re-establishing health or alleviating suffering. Where possible, these measures should be made the object of research, designed to evaluate their safety and efficacy. In all cases, new information should be recorded and, where appropriate, published. The other relevant guidelines of this Declaration should be followed.

**＊FOOTNOTE:** **Note of Clarification on Paragraph 29 of the WMA Declaration of Helsinki**

The WMA hereby reaffirms its position that extreme care must be taken in making use of a placebo-controlled trial and that in general this methodology should only be used in the absence of existing proven therapy. However, a placebo-controlled trial may be ethically acceptable, even if proven therapy is available, under the following circumstances:

Where for compelling and scientifically sound methodological reasons its use is necessary to determine the efficacy or safety of a prophylactic, diagnostic or therapeutic method; or

Where a prophylactic, diagnostic or therapeutic method is being investigated for a minor condition and the patients who receive placebo will not be subject to any additional risk of serious or irreversible harm.

**APPENDIX IV**

**RESEARCH PARTICIPANT INFORMED CONSENT FORM**

STUDY TITLE

**A Phase III, randomized, non-inferiority trial, to assess the efficacy and safety of Dihydroartemisinin+Piperaquine (DHA+PPQ, Artekin) in comparison with Artemether+Lumefantrine (A+L, Coartem) in children with uncomplicated *P. falciparum* malaria.**

**- Multicentre Study In Africa -**

**Study Protocol No: ST3073+ST3074 DM040010**

Please read the background information and informed consent form carefully. The background information explains your rights and our responsibilities to you. If you have any questions concerning the study please do not hesitate to ask any of the doctors. Before you decide, it is important for you to understand why the research is being done and what it will involve. You will be given a copy of this signed document (Informed Consent Form) to take home with you.

**YOU MUST KEEP THIS BACKGROUND INFORMATION WITH YOU THROUGHOUT THE STUDY PERIOD.**

**PURPOSE OF THE STUDY**

This research study is being done to learn more about the treatment of malaria. We are carrying out a research study to compare different medicines for the treatment of mild malaria. The medicines we are studying are: dihydroartemisinin-piperaquine (Artekin) and arthemeter-lumefantrine (Coartem). Both drugs are active against malaria. With this study we want to find out whether dihydroartemisinin-piperaquine can cure malaria as effectively and safely as arthemeter-lumefantrine. One thousand and five-hundred patients from five different African countries will participate in this study.

**HOW THE STUDY IS DONE**

The child under your care will be treated for malaria with one of the above study medicines. After the treatment, your child will be followed for 42 days to see if the malaria infection is completely cured. If your child is not completely cured by the study medicines, he/she will then be given treatment according to the standard practice in your country. The study medicine that your child will receive will be determined by a process of randomization. Randomization means that your child will receive one of the two medicines by chance. You are being asked to allow your child (child under your care in the case of a legal guardian), to participate in this study for up to 42 days or until such time as you or the study doctors decide that your child should no longer participate in the study. The study doctors may withdraw your child from the study for the following reasons:

1. If your child receives malaria medicines not prescribed by the study doctors
2. If your child develop a febrile illness in addition to malaria which makes it difficult for the doctors to tell which problem is causing the fever
3. If you chose to withdraw your consent to participate in the study
4. If we are unable to locate your child within 24 hours between days 1-14 and 48 hours between days 15-42.

The study may be discontinued by the sponsor at any time, and for any reason.

**PROCEDURES**

- 1. The study doctors will examine your child today.
  2. A blood sample will be collected. A small amount of blood will be taken by fingerprick to examine for malaria parasites, to measure the blood count, to store blood samples on filter paper for future laboratory tests that will not impact on the health care of your child.
  3. If the diagnosis of malaria is confirmed, and your child is eligible for the study, treatment with dihydroartemisinin + piperaquine (DHA+PPQ) or arthemeter + lumefantrine (A+L) will be given at the clinic during the first 3 days. This means that your child will be hospitalised the first 3 days of the study.
  4. At time of inclusion in the study an insecticide-treated bed net will be provided for your child. You will be asked to have your child sleeping under it to protect him/her from further malaria infections.
  5. You will be asked to return to the clinic at least 7 more times over the next month so that the success of the treatment can be judged. At each of the follow-up visits, your child will be examined by the study doctors and, a small amount of blood will taken by fingerprick to examine for malaria parasites and to save on filter paper.
  6. If case of missing appointment, the home health visitor will visit your child at your home to find out why you missed the appointment and bring your child to the clinic for assessment.
  7. If, at any time, the treatment given to your child does not seem to be working well, it will be changed to treatment according to the usual standard of care.
  8. There will be someone at the study clinic every day from 8:00 am to 5:00 pm and at night. You can come to the clinic for evaluation anytime that your child is ill during the next 28 days.
  9. For the haematology and biochemistry, there will be 4 blood samples: one before the first dose at D0 (Visit 1), the second one at D3 (visit 4), the third at D28 (Visit 8), i.e. one month after D0, and the last one at D42 (Visit 10) approx. 14 days after D28. Each sample will be of 2 mL and will be collected from an arm vein by an experienced nurse. Blood sampling may cause pain and swelling in addition small violet spots around the site of injection called hematoma could appear.

RISKS AND DISCOMFORTS

1. Side effects following treatment with the study medications could occur. Generally, side effects (nausea, headache, dizziness…) are expected to be mild and only short-lived.

Your child will be monitored closely after receiving treatment for malaria with the study medications for any possible side effects of the drugs and will receive appropriate medical care for any problem that happens during the course of the study.

1. Randomization: Your child will be assigned to a treatment group by chance. The treatment your child receives may prove to be less effective or to have more side effects than the other study treatments or than other available treatments. This will not be known until after the study is completed.
2. Severe malaria: Your child may develop malaria that is severe even after receiving treatment with study medications. If your child shows any evidence of severe malaria (including persistent vomiting, low blood (anaemia), convulsions, confusion, or coma) treatment with the usual standard of care will be given and your child will be referred for possible admission to hospital.
3. Blood draws: The risks of drawing blood from a fingerprick include temporary discomfort from the needle stick, bruising, skin infection, and fainting. The amount of blood removed will be too small to affect your child’s health.
4. Unknown Risks: The research treatments may have side effects that no one knows about yet. The researchers will let you know if they learn anything that might make you change your mind about your child’s participation in the study.
5. Confidentiality: Participation in research may involve a loss of privacy, but information about your child will be handled as confidentially as possible. Medical information related to malaria will be collected on your child, but only the people working on the study will see it. Anyone assigned to review this study will be granted direct access to your child's medical records, if necessary, for verification of the study procedures and data. Records will be kept as confidential as possible.

BENEFITS

1. The potential benefit to your child is that the treatment received may prove to be more effective than the other study treatments or than other available treatments, although this cannot be guaranteed.
2. Your child will receive clinical care from the medical officers and nurses of the project staff in the study clinic. This will include care for unscheduled sick visits.
3. The knowledge gained from this study will help your country in determining the best treatment for uncomplicated malaria.

COST/PAYMENT

After enrolment in the study, you will not be charged for clinic visits or treatment. Your child will not be paid for participation in the study. We will reimburse any transport costs incurred for clinic visits and meals will be provided when your child is admitted for observation and treatment administration. A insecticide-treated bed net, additional to that provided for your child, will be given to you at the end of the follow up.

**ALTERNATIVES TO PARTICIPATION**

Your child’s participation in this study is completely voluntary. If you decide that you do not want to participate in the study or decide to withdraw your child from the study at any time and for any reason, this will not affect your child’s care at the outpatient department, where standard care for all medical problems is available. During the study, you will be informed promptly of any new information that may influence your willingness to continue participation in the study.

**CONSEQUENCES OF WITHDRAWAL**

Should you or your study doctors decide to withdraw your child from the study before your child has finished the course of study medicines, then your child will receive the local standard treatment for malaria from the study team, but after the standard treatment has been given, medical care will no longer be provided by the study team. If the child is withdrawn from the study after completion of the course of study medicines, then no further care will be provided by the study team.

**USE OF THE RESULTS**

The findings from this study may be published in a medical journal. The study participants will not be identified by name. After the study is completed, you may request an explanation of the study results.

**TREATMENT AND COMPENSATION FOR INJURY**

If you are injured or have questions about injuries as a result of being in the study, please contact the doctors in the study clinic. The services at the public health facility will be open to you in case of any such injury.

**VOLUNTARY PARTICIPATION**

Participation in this study is entirely voluntary. You have the right to refuse your child’s participation or to withdraw at any point in this study without negative consequences or loss of benefits to which you and your child are otherwise entitled.

**implication of your SIGNATURE OR THUMBPRINT**

If you give consent for your child to participate in this study, you should sign or place your thumbprint in the consent form. Your signature or thumbprint below means that you understand the information given to you about your child’s participation in the study and in the consent form. You will also be asked to sign another copy of this informed consent form for documentation.

**CONSENT FORM**

**CONSENT FORM FOR PARTICIPATION IN RESEARCH PROJECTS**

**AND CLINICAL TRIALS**

**Study Title**

**A Phase III, randomized, non-inferiority trial, to assess the efficacy and safety of Dihydroartemisinin+Piperaquine (DHA+PPQ, Artekin) in comparison with Artemether+Lumefantrine (A+L, Coartem) in children with uncomplicated *P. falciparum* malaria.**

**- Multicentre Study In Africa -**

Principal Investigator: ___________________________________________________________

Address:______________________________________________________________________

Contact number_________________________________________________________________

I, …………………………………. mother/father/legal representative declare that I have understood the objectives and purposes of this study. I agree that my child may participate in this study.

I am aware that I can withdraw my child from the study at any time without any consequence to my child or to me.

Name of parent/legal representative

Signature or Thumbprint * of parent/ legal representative Date/Time

*If the parent or guardian is unable to read and/or write, an impartial witness should be present during the informed consent discussion. After the written informed consent form is read and explained to the parent or guardian, and after they have orally consented to their child’s participation in the trial, and have either signed the consent form or provided their fingerprint, the witness should sign and personally date the consent form. By signing the consent form, the witness attests that the information in the consent form and any other written information was accurately explained to, and apparently understood by, the parent or guardian, and that informed consent was freely given by the parent or guardian.

Name of Person Witnessing Consent (printed)

Signature of Person Witnessing Consent Date/Time

**APPENDIX V**

**Criteria for Severe Malaria/Danger Signs**

**Severe Malaria**

- Unarousable coma *(if after convulsion, > 30 min)*
- Repeated convulsions *(> 2 within 24 h)*
  - Severe anaemia *(Hb < 5.0 g/dL)*
  - Respiratory distress *(laboured breathing at rest)*

**Danger Signs**

- Recent convulsions *(1-2 within 24 h)*
- Altered consciousness  *(confusion, delerium, psychosis)*
- Lethargy
  - Unable to drink or breast feed
  - Vomiting everything
  - Unable to stand/sit due to weakness

**APPENDIX VI. PARTICIPANT SELECTION AND ENROLLMENT**

Children attending Health Facility (HF) aged between 3 and 59 months with history of recent fever

Send to laboratory

Screening thick blood smear

Negative smear or parasitaemia > 2000 < 200,000 parasites/µl

< 2,000 or > 200,000 /µl

Refer patient to clinician

Refer patient back to HF

No information collected

Complete Screening Form

Excluded Passed Initial Screening

Complete Informed Consent Form

Excluded

Refer patient back to HF

Save screening form only

Assign Study Number

Complete Case Report Forms

Refer to the Laboratory

Hb < 5.0 g/dl

Refer to the Investigator/Study Nurse

Admit at the clinic or manage as outpatients for 3 days

If a more carefull reading of the day 0 blood slide shows that parasite density outside required range: protocol violation. Continue follow up

Continue with scheduled follow-up.

**APPENDIX VII. PARTICIPANT SCREENING FORM**

**STUDY SITE CODE:________ SCREENING NUMBER: ________________**

SCREENING FORM

| **1. Names:** | **2. Date: *(dd/mm/yy)*** | **3.Weight (kg):** |
| --- | --- | --- |
| **4. Age: _________________months.** | **5. Gender: M _______ F_______** | |

| **SCREENING selection criteria**  ***Patients who are > 6 months of age and have a positive screening thick blood smear.*** | | |
| --- | --- | --- |
| **inclusion criteria** | **yes** | **NO** |
| 6. Fever (> 37.5°C) or history of fever in previous 24 hours |  |  |
| 7. Weight > 5 kg |  |  |
| 8. Ability to participate in 42-day follow-up. |  |  |
| **EXCLUSION CRITERIA** | **NO** | **YES** |
| 9. Participating in any investigational drug study during the previous 30 days? |  |  |
| 10. History of serious side effects to study medications  *If present, indicate drug / side effect:*   ________________________   __________________________   ________________________ |  |  |
| 11. Evidence of severe malaria / danger signs  *If “ YES” indicate criteria. If “NO”, leave blank.*   Unarousable coma *(if after convulsion, > 30 min)*   Repeated convulsions *(> 2 within 24 h)*   Severe anaemia *(Hb < 5.0 g/dL)*   Respiratory distress *(laboured breathing at rest)*   Recent convulsions *(1-2 within 24 h)*   Altered consciousness*(confusion, delerium,,psychosis)*   Lethargy   Unable to drink or breast feed   Vomiting everything   Unable to stand/sit due to weakness |  |  |
| 12. Evidence of concomitant febrile illness  *If “YES”, indicate illness. If “NO”, leave blank.*   Pneumonia/RTI  Measles   Otitis Media  UTI   Gastroenteritis  Other:_________________ |  |  |
| **INCLUSION CRITERIA** | **YES** | **NO** |
|  |  |  |
| 13. Provision of informed consent. |  |  |
| 14. Absence of persistent vomiting of study medications on day 0 |  |  |
| ***Complete at day 1.*** | | |
| 15. *P. falciparum* mono-infection |  |  |
| 16. Parasite density > 2000/µl and < 200,000/µl  *If “NO” specify density*  < 2000/µl  > 200,000/µl |  |  |

***If any of the responses fall into the shaded area, exclude the patient from the study***

**APPENDIX VIII. CRITICAL STEPS**

**Days 0 and 1**

Evaluate patient and complete Case Report form. Administer study medications.

**Day 2.**

Evaluate patient and complete Case Report Form. Administer study medications.

Collect thick blood smear and filter paper sample

**Day 3.**

Evaluate patient and complete Case Report Form. Collect thick blood smear and filter paper sample

**Day 7, 14, 21**. Evaluate patient and complete Case Report Form. Collect thick blood smear and filter paper sample

**Any Unscheduled Day (Day 4-27).**

Evaluate patient and complete Case Record Form. Collect thick blood smear with filter paper sample.

D2 Parasitaemia > D 0 parasitaemia

**ETF**

Treat with oral quinine

**Severe disease or danger signs**

Do urgent thick smear, FP sample and Haemoglobin

If patient has

1. Temperature > 37.5^0^C with parasitaemia or

2. Parasite count >25% Day 0 count.

**ETF**

Treat with oral quinine

**Days 28, 35 and 42.**

Evaluate patient and complete Case Report Form. Collect thick blood smear and filter paper sample.

**Severe disease or danger signs**

Do urgent thick smear, FP sample and Haemoglobin

Negative smear

**Continue study**

**at your discretion.**

Negative smear

**Continue study at your discretion.**

Positive blood smear

**ETF**

**Give/ refer for IV quinine.**

Positive blood smear

**LCF**

**Give/ refer for IV Quinine.**

**ACPR** No parasitaemia on days 28 or 42 irrespective of fever history or temperature.

**LPF** Parasitaemia D28 or D42 with temperature < 37.5^0^C without being ETF or LCF.

If patient has:

Temperature > 37.5^0^C with parasitaemia

**LCF**

Parasitaemia without fever

**LPF**

**APPENDIX IX** List of Investigators for the Mozambique Study site

**Investigator Principal**: Clara Menendez, Centro de Saude Internacional, Hospital Clinic/Universidade de Barcelona e Centro de Investigaçao em Saude de Manhiça

**Co-Investigators:**

Eusebio Macete: Centro de Investigaçao em Saude de Manhiça/Ministerio de Saude de Mozambique

Pedro Aide: Centro de Investigaçao em Saude de Manhiça

Quique Bassat: Centro de Saude Internacional, Hospital Clinic/Universitat de Barcelona i Centro de Investigaçao em Saude de Manhiça

Inacio Mandomando: Centro de Investigaçao em Saude de Manhiça

Montse Renom: Hospital Clínic-Universitat de Barcelona/CISM

Samuel Mabunda: Director do Programa de control de malaria, Ministerio de Saude de Moçambique

Betuel Sigaúque: Centro de Investigação em Saúde de Manhiça/Ministério de Saúde de Mozambique
