# Supplementary material for: Dihydroartemisinin-Piperaquine and Artemether-Lumefantrine for Treating Uncomplicated Malaria in African Children: A Randomised, Non-Inferiority Trial
Source: PLoS One. 2009 Nov 17;4(11):e7871. doi: 10.1371/journal.pone.0007871 (PMC2776302; doi:10.1371/journal.pone.0007871)
Supplement: Checklist S1 — CONSORT checklist (0.36 MB DOC) [file pone.0007871.s002.doc]

Dihydroartemisinin-Piperaquine versus Artemether-Lumefantrine for treating non-complicated malaria in African children: A randomised open-label, phase III, non-inferiority trial in five African countries.

**Authors**: Quique Bassat, Halidou Tinto, Modest Mulenga, Patrice Piola, Steffen Borrmann, Clara Menéndez, Michael Nambozi, Innocent Valéa, Carolyn Nabasumba, Philip Sasi, Antonella Bacchieri, Marco Corsi, David Ubben, Ambrose Talisuna, Umberto D’Alessandro

**Based on:**

Piaggio G, Elbourne DR, Altman DG, Pocock SJ, Evans SJ. Reporting of non-inferiority and equivalence randomized trials: an extension of the CONSORT statement. JAMA. 2006 Mar 8; 295(10):1152-60.1

**Item numbers:**

**Title and abstract (1)**: **How participants were allocated to interventions (eg, “random allocation,” “randomized,” or “randomly assigned”), *specifying that the trial is a non-inferiority or equivalence trial.*** The title reflects how patients were allocated to the intervention and that the trial is non-inferiority: “Dihydroartemisinin-Piperaquine versus Artemether-Lumefantrine for treating non-complicated malaria in African children: A randomised open-label, phase III, non-inferiority trial in five African countries”.

**Introduction: Background (2): Scientific background and explanation of rationale, including the rationale for using a non-inferiority or equivalence design.** This is addressed both in the introduction and the methods section.

**Methods: Participants (3)**: **Eligibility criteria for participants (detailing whether participants in the non-inferiority or equivalence trial are similar to those in any trial[s] that established efficacy of the reference treatment) and the settings and locations where the data were collected**. This is addressed in the methods section where all inclusion and exclusion criteria are detailed, under the heading “Study design, sites and concealment of patient allocation”, and in table 1 (online publication only), where relevant details of the sites involved in the trial are described.

**Interventions (4): Precise details of the interventions intended for each group, detailing whether the reference treatment in the non-inferiority or equivalence trial is identical (or very similar) to that in any trial(s) that established efficacy, and how and when they were actually administered**. This is addressed in the methods section where details of both treatments used are revealed, under the heading “Study design, sites and concealment of patient allocation”

**Objectives (5): Specific objectives and hypotheses, including the hypothesis concerning non-inferiority or equivalence.** Due to space limitations, the draft does not include the specific objectives and hypotheses, which are comprehensively defined in the protocol (page 16). Essentially:”The aim of the study is to determine whether a new formulation of DHA+PPQ is at least as good as (not inferior to) A+L for the treatment of African children with acute uncomplicated *P. falciparum* malaria and to assess its safety and tolerability.

**Primary Objective**

The primary objective of the study is to measure the Day 28, PCR corrected cure rates of Artekin and Coartem and demonstrate that:

- the cure rate of Artekin is non-inferior to that of Coartem (non-inferiority margin = 5%);

- the cure rate of Artekin is at least 90%.

This cure rate is defined as the proportion of patients with adequate clinical and parasitological response at Day 28.

**Secondary Objectives**

The secondary objectives of the study will be the between treatment comparison of:

- the uncorrected Day 28 cure rates of both drugs (risk for rescue therapy);

- the safety profiles of the two treatments;

- times of parasite clearance (PCT);

- time of fever clearance (FCT);

- gametocyte prevalences and densities on days 7, 14, 28, 42;

- haematological recovery (Haemoglobin (Hb) changes from day 0 to day 28 and day 42;

- cure rates at D42 (PCR corrected and PCR uncorrected).

**Outcomes (6): Clearly defined primary and secondary outcome measures, detailing whether the outcomes in the non-inferiority or equivalence trial are identical (or very similar) to those in any trial(s) that established efficacy of the reference treatment and, when applicable, any methods used to enhance the quality of measurements (eg, multiple observations, training of assessors)**. This is clearly defined in the Methods section, under the heading Outcome classification. Essentially: “The primary endpoint was the PCR-corrected adequate clinical and parasitological response (ACPR) at day 28; secondary efficacy outcomes included PCR-corrected cure rates at days 14 and 42, PCR-uncorrected cure rates at days 14, 28 and 42; parasite and fever clearance times, presence and clearance of gametocytes, and haemoglobin (Hb) recovery from baseline to day 28. All standard safety outcomes such as incidence of adverse events, changes from baseline on haematology and clinical chemistry parameters, ECG findings and vital sign variation during the study were also evaluated. Treatment outcome was analysed in two ways. The first was based purely on the standard definitions of early/late clinical and parasitological failure (World Health Organization)^2^. The second, agreed with the Data Monitoring and the Clinical Development Committees, was based on a pre-defined procedure (Table 2) [ONLINE PUBLICATION ONLY] complementing the WHO definitions with a set of rules allowing the evaluation of each individually randomised patient. Such an approach was defined as primary because it was deemed to be in line with the requirements of the most stringent regulatory authorities. All cases not strictly matching the WHO definitions and/or the described procedure were reviewed individually at the data review meetings”.

**Sample size (7): How sample size was determined, detailing whether it was calculated using a non-inferiority or equivalence criterion and specifying the margin of equivalence with the rationale for its choice. When applicable, explanation of any interim analyses and stopping rules (and whether related to a non-inferiority or equivalence hypothesis).** This is also addressed in the methods section, under the heading “sample size calculation”. Essentially the draft states: “This study was designed as a non-inferiority trial. According to previous studies, the PCR-corrected cure rate at day 28 for AL was estimated at around 90–92%^18^ in the modified ITT population and 94–95% in the PP population. Assuming 80% statistical power, a one-sided α level of 2.5%, and adopting an unequal 2:1 randomisation ratio, 1,500 patients (1000 DHA-PQP, 500 AL) would be needed to show that the difference of the PCR-corrected day-28 cure rate between DHA-PQP and AL was within -5%. This estimation accounted also for the rate of protocol violations (30% patient attrition rate in the PP). A blinded interim analysis, planned in the protocol, was carried out by the independent Data Monitoring Committee to verify the sample size assumptions”.

Table 2 (online publication only) defines the rules utilized for outcome classification.

**Randomization: Sequence generation (8): Method used to generate the random allocation sequence, including details of any restriction (eg, blocking, stratification)**. The draft only states “A randomisation list stratified by country was generated by an independent off site contract research organisation (CRO)”. The protocol states (page 27): The randomization list will be generated by MDS Pharma Services, using the plan procedure of SAS (Statistical Analysis System).

The full procedure followed to produce the randomization list is as follows:

Allocation ratio: 2:1 (2 for Artekin: 1for Coartem)

Number of centers: 5

Number of patients/block:

The first 80% of the patients of each center scheduled size were randomly assigned to blocks of 3, 6, 9 and 12 patients (first batch). The other 20% of patients were randomly assigned to blocks of 3 and 6 patients (second batch). No block was to be broken.

Also the order of block sizes was randomized.

- Batch 1: Block sizes (3, 6, 9 & 12) were randomly assigned in blocks of 8 (1:1:1:1).
- Batch 2: Block sizes (3 & 6) were randomly assigned in blocks of 4 (1:1).

**Allocation concealment (9): Method used to implement the random allocation sequence (eg, numbered containers or central telephone), clarifying whether the sequence was concealed until interventions were assigned**. The draft states: “A randomisation list stratified by country was generated by an independent off site contract research organisation (CRO), with each treatment allocation concealed in opaque sealed envelopes that were opened only after the patient’s recruitment”.

**Implementation (10): Who generated the allocation sequence, who enrolled participants, and who assigned participants to their groups**. The draft states: “A randomisation list stratified by country was generated by an independent off site contract research organisation (CRO), with each treatment allocation concealed in opaque sealed envelopes that were opened only after the patient’s recruitment”. It also states:”In order to minimise bias, treatment allocation was concealed until recruitment of the patient was completed. Both patient allocation to the different analysis populations and assessment of the primary end-point were made by staff blinded to the treatment assignment and before availability of the PCR results.”

**Blinding (masking) (11): Whether or not participants, those administering the interventions, and those assessing the outcomes were blinded to group assignment. When relevant, how the success of blinding was evaluated**. The study was open-label. The draft states “Both patient allocation to the different analysis populations and assessment of the primary end-point were made by staff blinded to the treatment assignment and before availability of the PCR results.” The draft also addresses this method’s limitations: Neither the patients nor the investigators were blind to the treatment administered and this may be considered as a limitation. However, guaranteeing adequate blinding through the use of the double-dummy technique would have resulted in a heavy treatment schedule for the patients and a greater difficulty in implementing the trial. Therefore, alternative techniques for reducing potential bias, such as concealment until final recruitment, blind allocation to analysis populations and assessment of the primary endpoint before availability of the PCR results, were employed”.

**Statistical methods (12): Statistical methods used to compare groups for primary outcome(s), specifying whether a 1- or 2-sided confidence interval approach was used. Methods for additional analyses, such as subgroup analyses and adjusted analyses**. This has been addressed in detail in the methods part, under the heading “statistical analysis”: Statistical analysis was done following an *a priori* established analytical plan. The intention-to-treat population (ITT) included all randomised patients having taken at least one dose of the study treatments. The per-protocol (PP) population included all randomised patients fulfilling the protocol eligibility criteria, having taken at least 80% of the study medication, completing the day-28 assessment and having an evaluable PCR in case of recurrent parasitaemia. All drop-outs and all patients with missing or non-interpretable PCR results were evaluated as failures in the ITT population and excluded from the PP population. In order to deal with a potential high drop-out rate, a modified ITT population, in which patients lost-to-follow-up for treatment-unrelated reasons were excluded, was also defined and identified as co-primary (together with the PP population). As the results in this population were extremely similar to the other two (ITT and PP), they are not reported here.

Efficacy analysis was based on a 97.5% (one-sided) confidence interval (CI) computed on the difference between the day 28 PCR-corrected cure rates of DHA-PQP and AL. To prove non-inferiority, the lower limit of this CI was to be within -5%, the non-inferiority margin. The Wald method (without continuity correction) was used to compute the CI, as this method was known to provide control of type I error around the nominal level for the 2:1 allocation, and also in the context of a hypothesis test of non-inferiority^17^. Secondary outcomes were assessed similarly. In addition, for sensitivity purposes, estimates of the failure rates were generated with the survival analysis, using the Kaplan-Meier method in which patients were censored when they were lost to follow-up, had a new infection or a non valid PCR result. When survival analysis was applied to the new infections, censoring of new infections was replaced by censoring of recrudescences. The Breslow-Day test, or logistic regression when the former was not applicable, was used to assess homogeneity across centres. For exploratory testing, categorical variables were compared using χ^2^ or Fisher’s exact test, and continuous variables using the Student t-test for independent samples. Cure rates were stratified by age (age groups: ≤12 months; >12 and ≤24 months; >24 months) though the study was not powered for proving efficacy within each age group.

Person-gametocyte-weeks rates for measuring gametocyte carriage and transmission potential were calculated as the number of weeks in which blood slides were positive for gametocytes divided by the number of follow-up weeks and expressed per 1,000 person-weeks.

**Results: Participant flow (13): Flow of participants through each stage (a diagram is strongly recommended). Specifically, for each group report the numbers of participants randomly assigned, receiving intended treatment, completing the trial protocol, and analyzed for the primary outcome. Describe protocol deviations from trial as planned, together with reasons**. Figure 1 addresses specifically the trial profile.

**Recruitment (14): Dates defining the periods of recruitment and follow-up**. Recruitment lasted exactly between August 16^th^ 2005 and July the 14^th^ 2006, although the draft only says “Between August 2005 and July 2006, a randomized open-label, multicentre clinical trial was carried…”

**Baseline data (15): Baseline demographic and clinical characteristics of each group**. This has been addressed in table 3.

**Numbers analyzed 16* Number of participants (denominator) in each group included in each analysis and whether “intention-to-treat” and/or alternative analyses were conducted. State the results in absolute numbers when feasible (eg, 10/20, not 50%)**. Regarding populations analyzed, the draft states: “The intention-to-treat population (ITT) included all randomised patients having taken at least one dose of the study treatments. The per-protocol (PP) population included all randomised patients fulfilling the protocol eligibility criteria, having taken at least 80% of the study medication, completing the day-28 assessment and having an evaluable PCR in case of recurrent parasitaemia. All drop-outs and all patients with missing or non-interpretable PCR results were evaluated as failures in the ITT population and excluded from the PP population. In order to deal with a potential high drop-out rate, a modified ITT population, in which patients lost-to-follow-up for treatment-unrelated reasons were excluded, was also defined and identified as co-primary (together with the PP population). As the results in this population were extremely similar to the other two (ITT and PP), they are not reported here”. Results have been expressed as suggested.

**Outcomes and estimation (17): For each primary and secondary outcome, a summary of results for each group and the estimated effect size and its precision (eg, 95% confidence interval). For the outcome(s) for which non-inferiority or equivalence is hypothesized, a figure showing confidence intervals and margins of equivalence may be usefu**l. Table 4a and 4b express the results for primary and secondary outcome as suggested.

**Ancillary analyses (18): Address multiplicity by reporting any other analyses performed, including subgroup analyses and adjusted analyses, indicating those pre-specified and those exploratory**. The other analyses performed are defined in the draft as follows: “Treatment outcome was analysed in two ways. The first was based purely on the standard definitions of early/late clinical and parasitological failure (World Health Organization)^2^. The second, agreed with the Data Monitoring and the Clinical Development Committees, was based on a pre-defined procedure (Table 2) [ONLINE PUBLICATION ONLY] complementing the WHO definitions with a set of rules allowing the evaluation of each individually randomised patient. Such an approach was defined as primary because it was deemed to be in line with the requirements of the most stringent regulatory authorities. All cases not strictly matching the WHO definitions and/or the described procedure were reviewed individually at the data review meetings”.

**Adverse events (19): All important adverse events or side effects in each intervention group**. This has been addressed specifically in table 6, and the two deaths occurred during the trial summarized individually. “Two deaths (one per group) occurred during the study. In Uganda, a 3 year-old girl died 24 h after commencing treatment with DHA-PQP. Sepsis or severe malaria was considered by the investigating clinician as the most likely cause. In Mozambique, an 18 month-old girl died 7 h after the first dose of AL. Severe malaria was considered the most likely cause of death, although other aetiologies such as sepsis, hypoglycaemia, heart conditions or bronco-aspiration could not be ruled out. Death was considered as possibly related to the study drug only in this case”.

**Comment: Interpretation (20): Interpretation of the results, taking into account the non-inferiority or equivalence hypothesis and any other trial hypotheses, sources of potential bias or imprecision and the dangers associated with multiplicity of analyses and outcomes**. This has extensively been addressed in the discussion.

**Generalizability (21): Generalizability (external validity) of the trial findings**. This has also been addressed in the discussion, putting emphasis for the importance of the data for the recommendation of DHA-PQP for its use in Africa, where the greatest burden of the disease is, and especially among African children.

**Overall evidence (22): General interpretation of the results in the context of current evidence**. Data regarding efficacy and safety of both treatments has been reviewed and cited throughout the site, and the results of this particular trial presented in this context.

1. Piaggio G, Elbourne DR, Altman DG, Pocock SJ, Evans SJ. Reporting of noninferiority and equivalence randomized trials: an extension of the CONSORT statement. JAMA. 2006 Mar 8; **295**(10):1152-60.
